# Type 1 vomeronasal receptor expression in juvenile and adult lungfish olfactory organ

**DOI:** 10.1186/s40851-023-00202-z

**Published:** 2023-03-10

**Authors:** Shoko Nakamuta, Yoshio Yamamoto, Masao Miyazaki, Atsuhiro Sakuma, Masato Nikaido, Nobuaki Nakamuta

**Affiliations:** 1 Laboratory of Veterinary Anatomy, Faculty of Agriculture, Iwate University, 3-18-8 Ueda, Morioka, Iwate 020-8550 Japan; 2 Department of Biological Chemistry and Food Sciences, Faculty of Agriculture, Iwate University, 3-18-8 Ueda, Morioka, Iwate 020-8550 Japan; 3 School of Life Science and Technology, Tokyo Institute of Technology, 2-12-1 Ookayama, Meguro-Ku, Tokyo, 152-8550 Japan

**Keywords:** Evolution, In situ hybridization, Lungfish, RNA-seq, Vomeronasal organ, Vomeronasal receptors

## Abstract

Lungfish are the most closely related fish to tetrapods. The olfactory organ of lungfish contains lamellae and abundant recesses at the base of lamellae. Based on the ultrastructural and histochemical characteristics, the lamellar olfactory epithelium (OE), covering the surface of lamellae, and the recess epithelium, contained in the recesses, are thought to correspond to the OE of teleosts and the vomeronasal organ (VNO) of tetrapods. With increasing body size, the recesses increase in number and distribution range in the olfactory organ. In tetrapods, the expression of olfactory receptors is different between the OE and VNO; for instance, the type 1 vomeronasal receptor (*V1R*) is expressed only in the OE in amphibians and mainly in the VNO in mammals. We recently reported that *V1R*-expressing cells are contained mainly in the lamellar OE but also rarely in the recess epithelium in the olfactory organ of lungfish of approximately 30 cm body length. However, it is unclear whether the distribution of *V1R*-expressing cells in the olfactory organ varies during development. In this study, we compared the expression of *V1Rs* in the olfactory organs between juveniles and adults of the African lungfish *Protopterus aethiopicus* and South American lungfish, *Lepidosiren paradoxa*. The density of *V1R*-expressing cells was higher in the lamellae than in the recesses in all specimens evaluated, and this pattern was more pronounced in juveniles than adults. In addition, the juveniles showed a higher density of *V1R*-expressing cells in the lamellae compared with the adults. Our results imply that differences in lifestyle between juveniles and adults are related to differences in the density of *V1R*-expressing cells in the lamellae of lungfish.

**Supplementary Information:**

The online version contains supplementary material available at 10.1186/s40851-023-00202-z.

## Background

Most tetrapods, with some exceptions such as birds and humans, possess two anatomically distinct olfactory organs: the olfactory epithelium (OE) and the vomeronasal organ (VNO). The OE and the VNO send axons to the main and accessory olfactory bulbs, respectively [1]. The OE and the VNO of tetrapods were formerly thought to have distinct functions: the OE detects general odorants, and the VNO detects pheromones. However, recent studies suggest that the OE and the VNO have partially overlapping functions and act synergistically [2–4].

There is no VNO in the fish olfactory organ. Ciliated olfactory receptor cells (ORCs) and microvillous ORCs are intermingled in the OE of teleosts [5], whereas the ciliated and microvillous ORCs are distributed separately in the OE and VNO of mammals [6]. It has been suggested that the ciliated and microvillous ORCs were intermingled in the OE of common ancestors, but they have separated during evolution, giving rise to the mammalian OE and VNO containing ciliated and microvillous ORCs, respectively [5, 7]. The African clawed frog *Xenopus*, an amphibian that spends its entire life under water, has two main olfactory organs: the OE, which contains mainly ciliated ORCs, and the middle chamber epithelium, which contains both ciliated and microvillous ORCs. In addition, *Xenopus* has a VNO, which contains microvillous ORCs [8, 9]. The ultrastructural features of the *Xenopus* olfactory organs represent an intermediate step to the separated distribution of ciliated and microvillous ORCs.

Major olfactory receptor families of vertebrates, including odorant receptors (ORs), trace amine-associated receptors (TAARs), and type 1 and type 2 vomeronasal receptors (V1Rs and V2Rs), are G protein-coupled receptors; ORs and TAARs are coupled with Golf, V1Rs with Gi2, and V2Rs with Go [10, 11]. The signal transduction of ORs and TAARs involves cyclic nucleotide-gated channel alpha 2 [11, 12], whereas that of both V1Rs and V2Rs involves transient receptor potential channel 2 (TRPC2) [13]. From teleosts to mammals, ORs and TAARs are generally expressed by ciliated ORCs, and the V1Rs and V2Rs are expressed by microvillous ORCs [3, 5, 6, 11, 14]. In teleosts, all of the olfactory receptor families are expressed in the OE containing both ciliated and microvillous ORCs. In tetrapods, the olfactory receptor expression is segregated between the OE and VNO. In mammals, the ORs and TAARs are expressed in the OE containing ciliated ORCs, whereas the V1Rs and V2Rs are expressed in the VNO containing microvillous ORCs [15]. In addition, in amphibians, V1Rs are expressed in the OE and middle chamber epithelium, but not in the VNO [16].

The OE and VNO are classified as the main and accessory olfactory organs, respectively. Other than tetrapods, sea lamprey (Cyclostomata) and *Polypterus* (basal actinopterygians) have accessory olfactory organs [17–20]. However, in terms of the fine structure of ORCs and the expression of olfactory receptors, the accessory olfactory organs of sea lamprey and *Polypterus* are identical to the main olfactory organ (OE), although they are anatomically separated from the OE.

Lungfish are members of the Sarcopterygii and most closely related to tetrapods. They have two types of sensory epithelia in the olfactory organ: the lamellar OE covering the lamellar surface and the recess epithelium (RecE) contained in recesses at the base of lamellae. The lamellar OE and RecE are considered to correspond to the teleost OE and tetrapod VNO, respectively, based on the fine structure of ORCs, G-protein expression, and axonal projections to the olfactory bulbs [21–26]. In addition, the number and distribution of recesses vary among differently sized individuals of the African and South American lungfish [25, 27]. Also, in two species of the African lungfish, *Protopterus annectens* and *P. amphibius*, *V1R*-expressing cells are distributed mainly in the lamellar OE and slightly in the RecE [28]. However, it is unclear whether the distribution of *V1R*-expressing cells varies among individuals of different body sizes. In this study, we compared *V1R* expression in the lungfish olfactory organ among individuals of different body sizes to determine whether the distribution of *V1R*-expressing cells changes with growth stage.

## Materials and methods

### Animals

All procedures were approved by the local Animal Ethics Committee of Iwate University. The African lungfish *P. aethiopicus* and South American lungfish, *L. paradoxa*, were purchased from commercial suppliers. The fishes were anesthetized with tricaine methanesulfonate and euthanized by decapitation. Information pertaining to the animals is shown in Table 1. Juvenile and adult individuals of each lungfish were used. According to Mlewa and Green (2004) [29] and Jorgensen and Joss (2010) [30], *P. aethiopicus* individuals over 43 cm in body length (BL) reach sexual maturity. Thus, *P. aethiopicus* #1 (BL 50 cm) and *L. paradoxa* #1 (BL 65 cm) were regarded as adults, whereas *P. aethiopicus* #2–4 and *L. paradoxa* #3 (BL 35 cm or less) were regarded as juveniles [29, 30]. Also, we confirmed during dissection whether they had functional genital organs or not.

**Table 1:** Animals

	Animal No	Total body length (cm)	Body weight (g)	Sex	Application
*P. aethiopicus*	1	50.0	349.0	F	ISH (left)/RNA extraction (right)
	2	35.0	150.6	M	Dice CT
	3	31.5	100.0	unknown	ISH
	4	34.0	118.3	F	SEM
*L. paradoxa*	1	65.0	994.5	F	RNA extraction (left)/ISH (right)
	3	18.5	18.6	M	ISH

*ISH *in situ hybridization; *Dice CT* Diffusible iodine-based contrast-enhanced computed tomography; *SEM* Scanning Electron Microscopy

For histological examination, olfactory organs were dissected from the heads and fixed in 4% paraformaldehyde in 0.1 M phosphate buffer (PB, pH 7.4). The specimens were cryoprotected in a sucrose gradient (10%, 20%, and 30% in 0.1 M PB), embedded in O.C.T. compound (Sakura Finetek, Tokyo, Japan), and sectioned sagittally using a cryostat. Sections (20 µm in thickness) were thaw mounted on MAS-coated slides (Matsunami, Osaka, Japan), air-dried, and processed for hematoxylin–eosin staining, immunohistochemistry, and in situ hybridization.

### Diffusible iodine-based contrast-enhanced computed tomography (diceCT)

The diffusible iodine-based contrast-enhanced computed tomography (diceCT) procedure followed a previous study [31]. The olfactory organ was fixed in 4% paraformaldehyde in 0.1 M PB (pH 7.4) and stained with an aqueous solution of Lugol’s iodine (I_2_KI), 1% I_2_ and 2% KI in deionized water, for several days at room temperature (RT). Specimens were scanned using a microfocus X-ray CT system, inspeXio SMX-90CT (Shimadzu Corporation, Kyoto, Japan). The diceCT data were analyzed and visualized using VGStudio MAX software (System Create, Osaka, Japan).

### Scanning Electron Microscopy (SEM)

For Scanning Electron Microscopy (SEM), the olfactory organ was fixed in 2.5% glutaraldehyde in 0.1 M PB (pH 7.4) and postfixed in 1% osmium tetroxide. The dehydrated specimens were dried with t-butyl alcohol using a freeze dryer, ES2030 (Hitachi, Tokyo, Japan). The specimens were coated with osmium and examined by SEM (JSM7001F; JEOL, Tokyo, Japan).

### Immunohistochemistry

Immunohistochemistry using a rabbit anti-neural cell adhesion molecule (NCAM) antibody (AB5032, Millipore, Burlington, MA) and rabbit anti-Gαo antibody (551, MBL, Tokyo, Japan) was performed using olfactory organ sections from lungfish as described previously [23, 28]. Sections were incubated with each primary antibody overnight at 4°C, washed, and then incubated with a secondary antibody, Alexa Fluor 488-donkey anti-rabbit IgG (A21208, Thermo Fisher Scientific, Waltham, MA) for 2 h at RT. The sections were mounted in VectaShield mounting medium with DAPI (H-1200, Vector Laboratories, Burlingame, CA).

### Identification of lungfish V1R genes

Total RNA extracted from the olfactory organs using the ISOGEN reagent (Nippon Gene, Tokyo, Japan) was analyzed by RNA sequencing as described previously [28]. Briefly, the NovaSeq 6000 instrument was used (Illumina, San Diego, CA, USA), and reads were deposited in the DDBJ Sequence Read Archive (accession No. DRA015344 for *P. aethiopicus* and DRA015345 for *L. paradoxa*). De novo transcriptome assembly was performed using Bridger software [32]. We then used FATE (https://github.com/Hikoyu/FATE/commits/master) to search the V1R genes for assembled contig sequences. The V1R amino-acid sequences of two lungfish obtained in this study were aligned using MAFFT (ver. 7.475) [33] to those of a previous study [28]. Phylogenetic trees were constructed using the maximum likelihood method employing the best-fitting model of RAxML (ver. 8.2.12) [34], as estimated using the modeltest function of MEGA X [35]. Rapid bootstrap analyses were performed using 1,000 replicates to assess node reliability. The phylogenetic tree was visualized with FigTree (ver. 1.4.4; http://tree.bio.ed.ac.uk/software/figtree/).

### Reverse transcription PCR and gene cloning

The nucleotide sequences of primers specific to the V1R genes of *P. aethiopicus* and *L. paradoxa* are shown in Table 2. cDNA was synthesized from total RNA derived from the olfactory organs using oligo dT primers and ReverTra Ace (Toyobo, Osaka, Japan) according to the manufacturer’s protocol. PCR was performed using the cDNA as the template together with Ex Taq (Takara, Shiga, Japan). The PCR products were analyzed by 1.5% agarose gel electrophoresis.

**Table 2:** Primers for *V1Rs*

Probe name	Target V1R gene	Product size (bp)	Forward primer (5 > 3)	Reverse primer (5 > 3)
Paeth01	*P. aethiopicus V1R23 (ancV1R)*	597	CCCACAGTTAGCTGGCGTAA	GGTTTTGGCATGCCTCATGG
Paeth02	*P. aethiopicus V1R52*	403	CATTGGTTTGACCTGCCTGC	CTCTGCTCCAGCTTCCTGAC
Paeth03	*P. aethiopicus V1R53*	566	AGCCTAGCATGCTCAAACCT	ACCACCATCTTGGATGCCTG
Paeth04	*P. aethiopicusV1R55*	551	TGCTGTTGGCCTTGCAAGTA	TTGCCACAGCCATAAGGACT
Paeth05	*P. aethiopicusV1R69*	599	TGCTAAGCTGCTTCCAGTGT	AGAGTGGCAAGTCACTGCAT
Paeth06	*P. aethiopicusV1R71*	658	CTTCTGACTGGGGGTGTTCC	CCAAGGACAGAAAATGCCGC
Paeth07	*P. aethiopicusV1R83*	596	ACTTGCCAACCCACCAAGAA	GAAATGCAACGTCACGAGCA
Paeth08	*P. aethiopicusV1R94*	563	CGTGTTTGTCGAGCGATGTC	GCAAAGAAGACACGGGCATC
Paeth09	*P. aethiopicusV1R103*	545	CTTTTCACGCTGGGACTTCC	GTGACAACAGTCTTGGCAGC
Paeth10	*P. aethiopicusV1R111*	543	GGGGCAAACCTGTTACTCCT	TGCTTGTAGCTCTGCTGTGG
Paeth11	*P. aethiopicusV1R116*	523	CGAGAGGCATTCCTGAACCA	TTAGCTGCCTGACCTTCTGC
Paeth12	*P. aethiopicusV1R119*	402	GACAAGTACTGGTGTTCTGGGT	TAAAGGAGCAGGCCACAACA
Paeth13	*P. aethiopicusV1R136*	477	TGATCCTTTGCAACCTGGGA	ACAAAATGTTGCTGCTGGCC
Paeth14	*P. aethiopicusV1R140*	589	CCGTGTTTTTCGAGCGATGT	AGGACTGACAGCAGCATACA
Paeth15	*P. aethiopicusV1R141*	513	CCAGAGGAATGCCACAGACA	CCCTGGCTTCAGCTGAAACA
Paeth16	*P. aethiopicusV1R144*	432	CACTGAACTGGCAGGGACAA	ATCAGGTCACGGGCAAAACT
Paeth17	*P. aethiopicusV1R148*	645	TCAGAGCTGTCAGTGGCAAA	CCGTGACACTGATGCCTGAT
Paeth18	*P. aethiopicusV1R159*	393	CAAGTACTGGAGTCTTGGGCA	GCAGGCCACAATGCATAACC
Paeth19	*P. aethiopicusV1R160*	695	TGGAAACATCACATCCGGCA	TGCTTGCTTCTCTGCTGTGA
Paeth20	*P. aethiopicusV1R166*	525	TACCCGAGGTCTTCCAGCAA	GCTGCTTTCACCTCTACAGC
Paeth21	*P. aethiopicusV1R198*	410	GTAGTAAGCGGCATCCCTGG	ACAGTGTACATTGGTGGGCT
Paeth22	*P. aethiopicusV1R208*	527	GGTTGTGCTGACAGTAGGCA	CTTGGGCTTCTGCACTGTTC
Paeth23	*P. aethiopicusV1R213*	549	ATGGTTGCTTTGCTGTCACG	TACAACCGACTTTGCAGCCT
Paeth24	*P. aethiopicusV1R218*	539	AGCTTCACAAAAGGGGCCAT	GCAAAGCCGTTCACCTGAAA
Paeth25	*P. aethiopicusV1R227*	520	CAAGAGGGGTTCCAGACTGT	GCTGCTCTGTTCTCTGCTGT
Paeth26	*P. aethiopicusV1R257*	305	CTCTGTGTGCTTGCTATGGC	ACTGTTTTTGCTGCTTGGCC
LP01	*L. paradoxa V1R20 (ancV1R)*	599	TACTGTTAGCTGGCGCAACA	TCTGCGTTTGGGGATTCCTC
LP02	*L. paradoxa V1R59/L. paradoxa V1R60*	620	AATGAGCTGCCCCAAACTGA	AGGTGACAACAGTTCGCGTA
LP03	*L. paradoxa V1R64*	640	ACAGTTACTGGAGCTGTGGG	TCTCTGCACTGTTCTCCAGC
LP04	*L. paradoxa V1R65*	543	ACCTGTCAACAGCAAACCTGA	TTGCTGCTTGACTCTCTGCA
LP05	*L. paradoxa V1R70*	525	TGCAAGAGGAGTGCCACAAA	GATTTTGCTGCCCTGGCTTC
LP06	*L. paradoxa V1R80*	552	ACCAGCAAACCTCACCATCA	ATGTAGCTGCTGGCAAGTGT
LP07	*L. paradoxa V1R89*	554	TTGCTGTCCGGAGTAAACCT	GCTGCTTGGCTTTCTGCATT
LP08	*L. paradoxa V1R92/L. paradoxa V1R93*	490	GGATCAGTGTCCTGGACAGC	TGAGGTCACGGCCAAAAAGA
LP09	*L. paradoxa V1R99*	652	CAGGTCTCTCTGGGGACTGA	AGGCAAAGTGTTGAGGCAGT
LP10	*L. paradoxa V1R103/L. paradoxa V1R104*	510	ACTTGGCCATCACTGGATCC	CCACCATGAGATCTCGGCTG
LP11	*L. paradoxa V1R120/L. paradoxa V1R121*	439	TCACATCCCACCTTGCTTTT	ATTACAGCATCACGCCCTGT
LP12	*L. paradoxa V1R127*	707	CTGCCCATGGTCTTCTCCAA	AATGGGGTCTCACCTGTTGC
LP13	*L. paradoxa V1R130*	556	TCCTGCCAACATTGCCATCT	AAAAAGGATTGCTGCGCTGG
LP14	*L. paradoxa V1R139*	412	TATCACGCGGCATGGCTATT	GACTCGGTGCGATCCTTCAT
LP15	*L. paradoxa V1R142*	524	AGTGTGTGAGTGTCAGTGCA	TGCAGCATAGCACATCGAGA
LP16	*L. paradoxa V1R172*	540	GAGCTGCTTCCAGTATGCCA	ACTGAAGCATAGCACGTGGA

For RNA probe synthesis, each PCR product was subcloned into the pCRII-TOPO vector using the TOPO TA Cloning Kit Dual promotor (Thermo Fisher Scientific). Next, the sequence of each clone was verified. Closely related V1R genes cannot be distinguished due to their high sequence identity, so some probes (LP02, LP08, LP10, and LP11) were expected to detect multiple V1R genes (Table 2).

### In situ hybridization

Digoxigenin (DIG)-labeled sense and antisense RNA probes were synthesized from plasmids linearized with restriction enzymes using the DIG RNA Labelling Kit (SP6/T7) (Sigma-Aldrich, St. Louis, MO). After being treated with DNase and EDTA, probes were precipitated with ethanol, dissolved in water, aliquoted, and stored at − 80°C until use. Sections were fixed in 4% (w/v) paraformaldehyde in 0.1 M PB for 10 min at RT, treated with 40 µg/mL proteinase K for 15 min at 37°C, and immersed in 0.1% (v/v) acetic anhydrate in the acetylation buffer for 15 min at RT. Hybridization was performed in the hybridization buffer ISHR7 (Nippon Gene) overnight at 55°C. Post-hybridization washing was performed in formamide/2 × saline-sodium citrate (SSC) for 1 h and 0.1 × SSC for 2 h at 55°C. The sections were incubated with anti-DIG antibody coupled to alkaline phosphatase (Roche Diagnostics, Basel, Switzerland) for 2 h at RT, and color was developed using NBT/BCIP stock solution (Roche) for signal detection.

### *V1R*-expressing cell density

In sections subjected to in situ hybridization using each *V1R* probe, labeled cells in the lamellae and recesses were counted, and their areas were measured using ImageJ software (https://imagej.nih.gov/ij/) as described previously [28]. The number of labeled cells in the lamellae or recesses was divided by the respective area to calculate the density of labeled cells for each probe (number of labeled cells per 1 mm^2^).

## Results

**Fig. 1:**
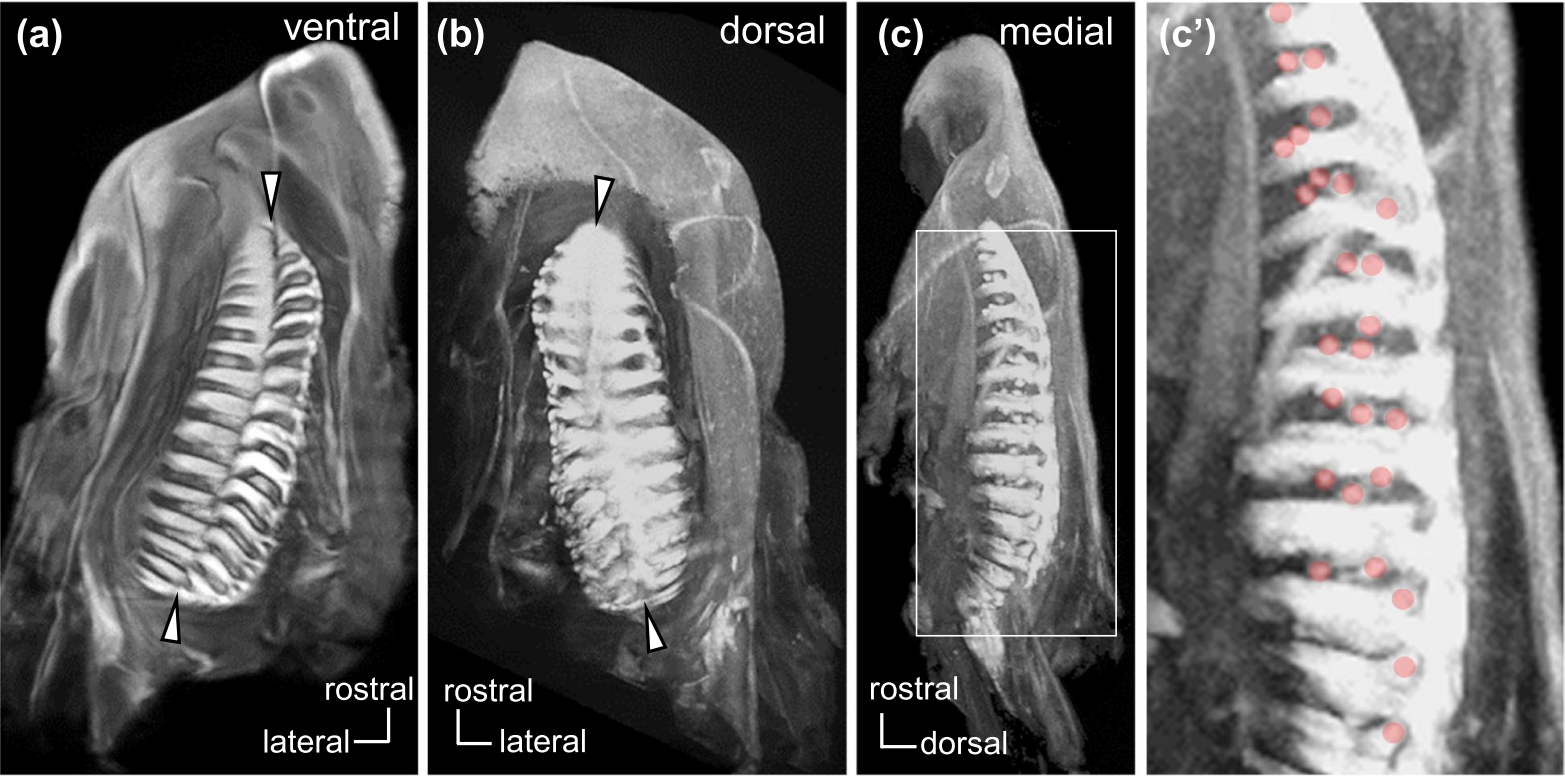
Diffusible iodine-based contrast-enhanced computed tomography images of the olfactory organ in *P. aethiopicus*. The right olfactory organ viewed from the ventral (**a**), dorsal (**b**) and medial (**c**) aspects. Higher magnification view of (**c**) is shown in (**c’**). Lamellae were arranged on the medial and lateral sides of the midline raphe (arrowheads). Recesses were abundantly present at the base of lamellae. The recesses are highlighted by red-shaded circles in (**c’**)

**Fig. 2:**
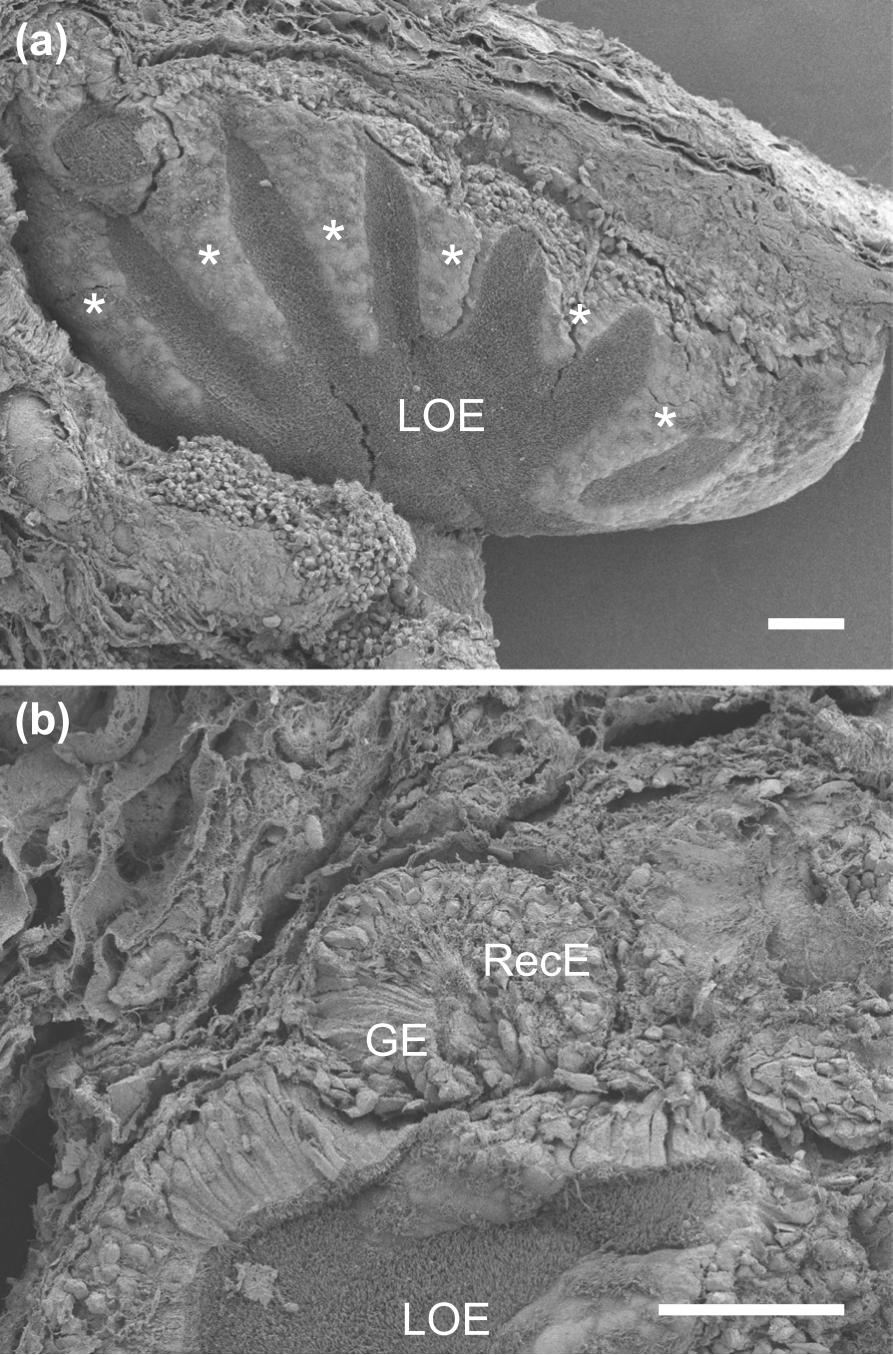
Scanning electron microscopy of a lamella cut out from the olfactory organ of *P. aethiopicus*. (**a**) Surface of the lamella covered with the non-sensory epithelium (asterisks) and lamellar OE (LOE). (**b**) Higher magnification view of a recess at the base of lamella consisting the recess epithelium (RecE) and glandular epithelium (GE). Scale bars: 100 µm

**Fig. 3:**
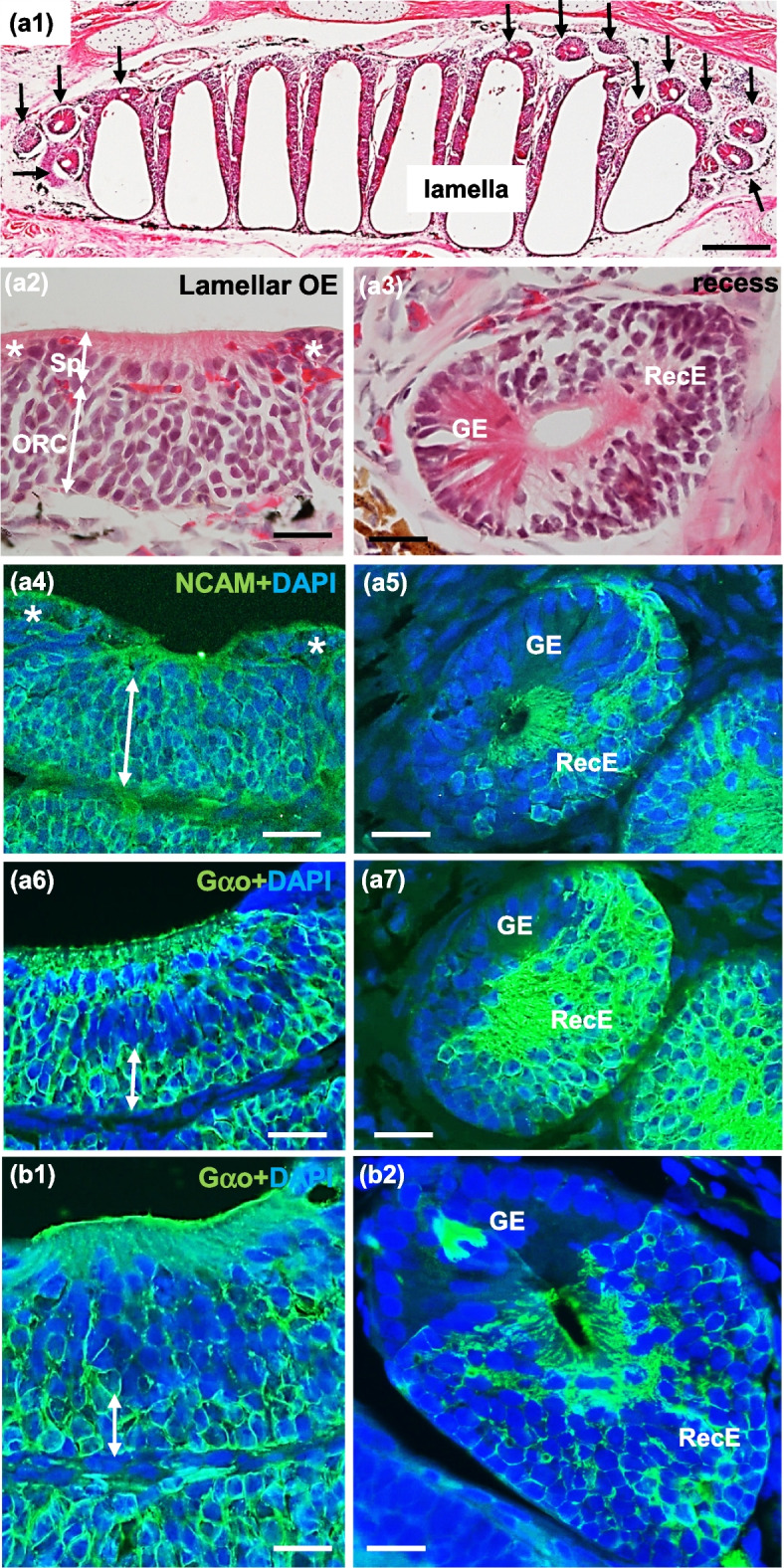
Structure of the olfactory organ of *P. aethiopicus* (**a1**-**a7**) and *L. paradoxa* (**b1**-**b2**). (**a1**) A sagittal section of the olfactory organ stained with hematoxylin–eosin, showing lamellae suspending from the dorsal wall. Dorsal is top, rostral is left. Recesses are found at the base of lamellae (arrows). (**a2**) The lamellar OE stained with hematoxylin–eosin showing round nuclei of the olfactory receptor cells (ORC) in the basal to middle layer, and oval nuclei of the supporting cells (Sp) in the upper layer. The lamellar OE and non-sensory epithelium (asterisks) are arranged alternately. (**a3**) A recess stained with hematoxylin–eosin consisting of the recess epithelium (RecE) which contains several layers of cells with round nuclei, and the glandular epithelium (GE) which contains eosinophilic cytoplasm and basally located round nuclei. (**a4**) The layer of ORCs immunopositive for NCAM and the non-sensory epithelium (asterisks) immunonegative for NCAM. (**a5**) The RecE immunopositive for NCAM and the GE immunonegative for NCAM. (**a6** and **b1**) The basal layer of lamellar OE is immunopositive for Gαo. (**a7** and **b2**) Most ORCs in the RecE are immunopositive for Gαo. GE is immunonegative for Gαo. Scale bars: 500 µm in (**a1**), 50 µm in (**a2**–**a7**, **b1**, **b2**)

In the olfactory organ of lungfish, lamellae were arranged on the medial and lateral sides of the midline raphe, and recesses were abundant at the base of lamellae (Fig. 1). The surface of lamellae was covered with lamellar OE and non-sensory epithelium (Fig. 2a). The recesses consisted of RecE and glandular epithelium (GE) (Figs. 2b, 3a1, a3). The overall histological and histochemical features of the olfactory organ were shared by *P. aethiopicus* (Fig. 3a1–a7) and *L. paradoxa* (Fig. 3b1,b2): In the lamellar OE, nuclei of the ORCs were located in the basal to middle layer, and those of supporting cells were located in the superficial layer (Fig. 3a2). The lamellar OE and RecE were immunopositive for the neuronal marker NCAM and thus distinguished from the non-sensory epithelium immunonegative for NCAM (Fig. 3a4, a5). The ORCs located in the basal layer of the lamellar OE and the majority of ORCs in the RecE were immunopositive for Gαo, an α subunit of the G protein coupled to V2Rs (Fig. 3a6, a7, b1, b2). In *P. aethiopicus* and *L. paradoxa*, Gαo-expressing ORCs were distributed in the RecE and basal layer of the lamellar OE: these histological and histochemical characteristics were shared between adults and juveniles, and were consistent with those reported in the olfactory organs of *P. annectens* and *P. dolloi* [21, 22].

**Fig. 4:**
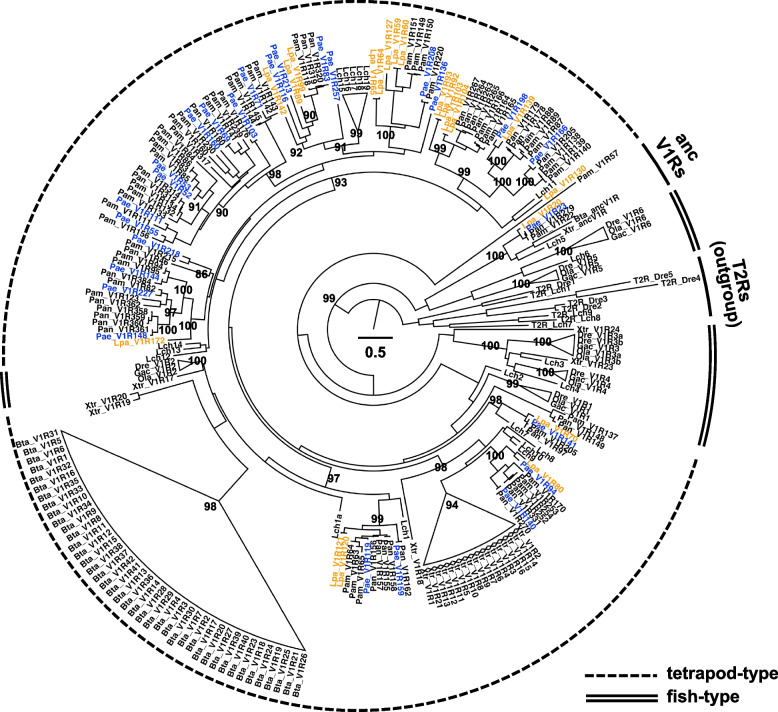
Phylogenetic tree of V1R genes of four lungfish. The names of the sequences are as follows: Pae, *P. aethiopicus*; Lpa, *L. paradoxa*; Pan, *P. annectens*; Pam, *P. amphibius*; Ola, medaka; Gac, stickleback; Dre, zebrafish; Lch, coelacanth; Xtr, tropical clawed frog; and Bta, cow. Newly identified lungfish V1Rs are indicated in blue (*P. aethiopicus*) and orange (*L. paradoxa*). Scale bars indicate the number of amino acid substitutions per site. T2Rs (bitter-taste receptors) were used as the outgroup. Bootstrap values higher than 80 were shown only for major nodes of the phylogenetic tree. Tetrapod-type and fish-type V1Rs are indicated by dotted and double lines, respectively

V1R genes expressed in the olfactory organs of two species of lungfish were identified by RNA-seq analysis. We found 26 V1R genes in *P. aethiopicus* and 20 in *L. paradoxa*, of which the nucleotide sequences are shown in Supplementary Data S1 and S2. The phylogenetic tree of four lungfish and six representative vertebrates suggested that the V1Rs can be divided into two major groups, fish-type and tetrapod-type (Fig. 4), which is consistent with previous studies [28, 36]. Except for ancV1R, all of the four lungfish V1Rs were of the tetrapod-type, and monophyly was supported by the near-maximum bootstrap probability (Fig. 4). Notably, the lungfish V1Rs tend to form clusters in each species, suggesting an increase via species-specific gene duplications.

**Fig. 5:**
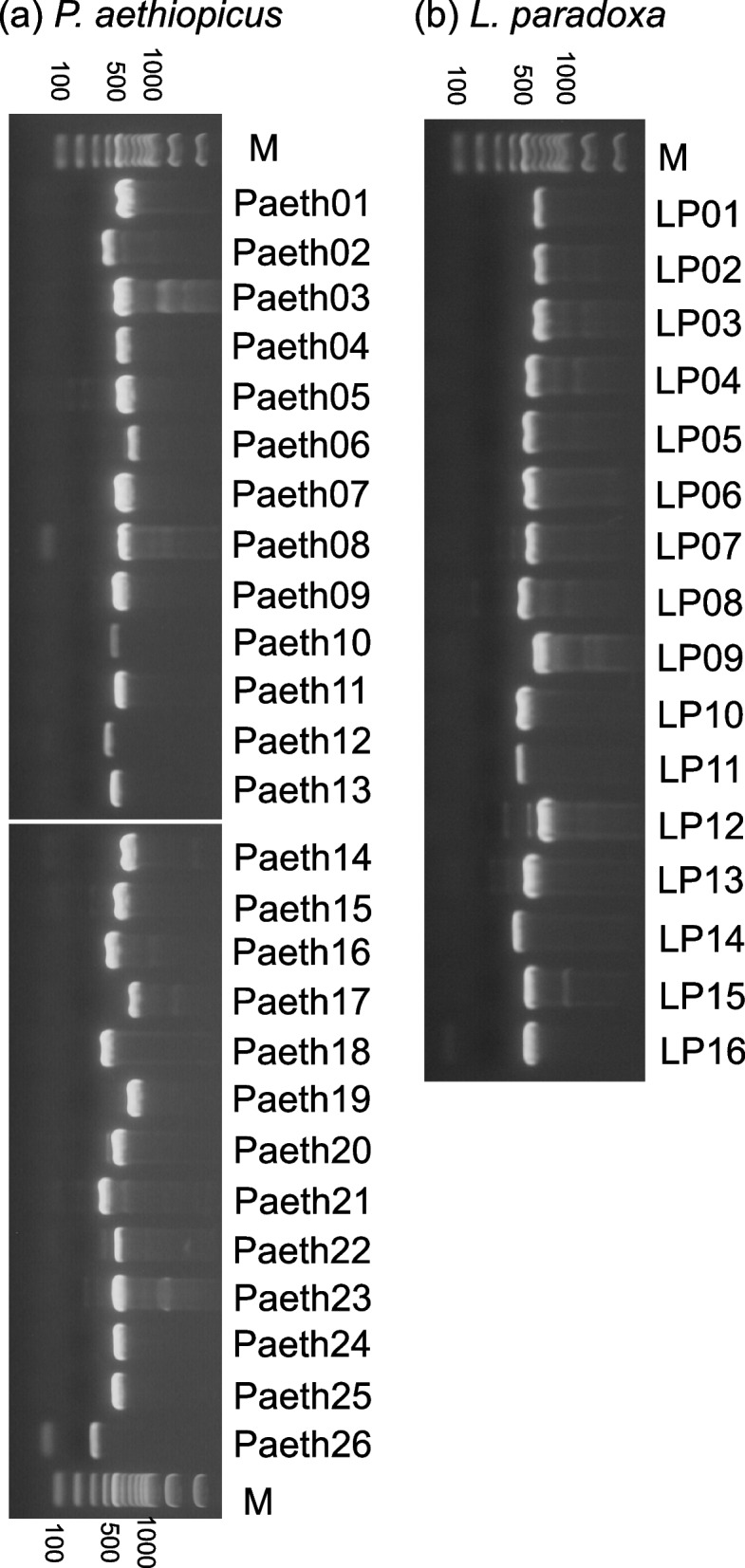
RT-PCR analysis for the expression of *V1Rs* in the olfactory organ of *P. aethiopicus* (**a**) and *L. paradoxa* (**b**). All *V1Rs* analyzed in this study are expressed in the olfactory organ. Lane M: FastGene100bp DNA ladder

Reverse-transcription PCR of the adult olfactory organs using the primers shown in Table 2 resulted in DNA fragments of the expected size for all V1R genes, indicating that these *V1Rs* are expressed in the lungfish olfactory organ (Fig. 5). Next, using probes prepared from these PCR products, in situ hybridization was conducted to visualize *V1R* expression in the lungfish olfactory organ.

**Fig. 6:**
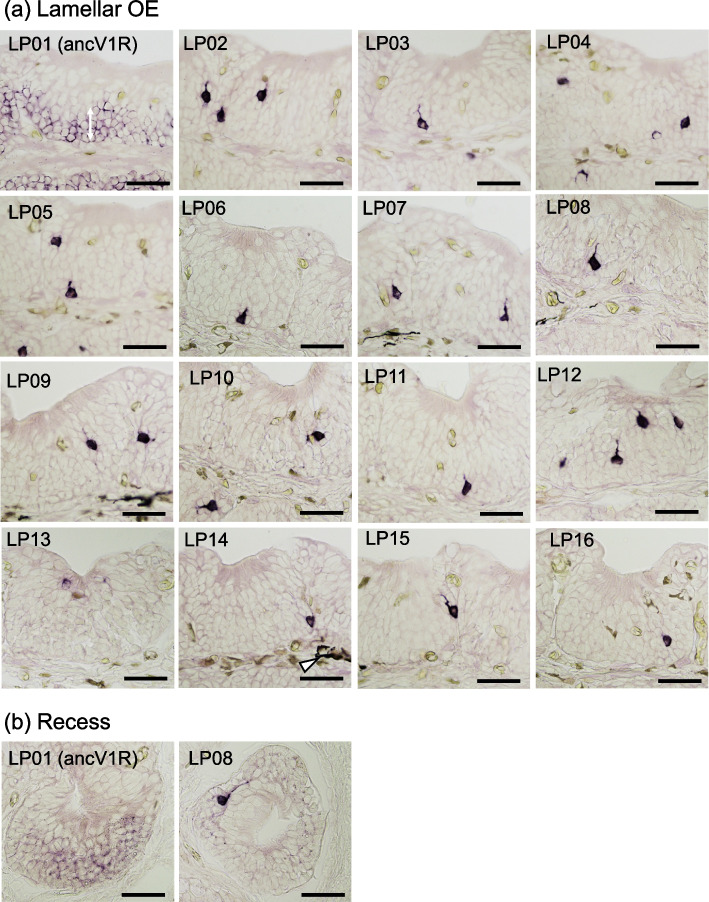
*V1R* expression in the lamellar OE (**a**) and recess (**b**) of *L. paradoxa*. **a** Signals for *ancV1R* in the basal to middle layer of the lamellar OE (double-headed arrow in LP01) and punctate signals for the other *V1Rs* (LP02–LP16) in the basal to middle layer of the lamellar OE. Black spots around the epithelium (for example, arrowhead in LP14) are melanophore aggregations. **b** Signals for *ancV1R* in most ORCs of the RecE and a signal for LP08 in the RecE. Scale bars: 100 µm

*V1R* expression in the olfactory organ of adult *L. paradoxa* and *P. aethiopicus* is shown in Fig. 6 and Supplementary Fig. S1, respectively. In both lungfishes, *ancV1Rs* (Paeth1, LP1) were expressed in RecE and the basal layer of lamellar OE (Fig. 6, Supplementary Fig. S1). This is consistent with the expression pattern of *ancV1R* in the *P. annectens* olfactory organ [20]. Aside from *ancV1R*, signals for all *V1Rs* were detected in the lamellar OE in adult *L. paradoxa* (Fig. 6, Table 3). In adult *P. aethiopicus,* signals for all probes except Paeth24 were detected in the lamellar OE (Supplementary Fig. S1, Table 4). By contrast, no signal was detected for any probe except LP8 in the recesses of *L. paradoxa* (Fig. 6), and no signal was detected for any probe in the recesses of *P. aethiopicus* (not shown). The lack of signals in the recesses by single probe in situ hybridization suggests the presence of very few or no *V1R*-expressing cells in the recesses. To address this issue, in situ hybridization was conducted using a mixture of all *V1R* probes except *ancV1R* to examine the number and distribution of *V1R*-expressing cells. In *P. aethiopicus* (Supplementary Figs. S2, S3) and *L. paradoxa* (Supplementary Figs. S4, S5), signals were distributed mainly in the lamellar OE, but slightly in the recesses, in both juveniles and adults (Table 5). In *P. aethiopicus*, the densities of *V1R*-expressing cells in the lamellae and recesses were 36 and 0.75 cells/mm^2^ in the adult vs. 61 and 0.09 cells/mm^2^ in the juvenile, respectively. Thus, the density of *V1R*-expressing cells was approximately 48-fold higher and 670-fold higher in the lamellae than in the recesses in the adult and the juvenile, respectively (Table 5). In *L. paradoxa,* the densities of *V1R*-expressing cells in the lamellae and recesses were 15 and 0.33 cells/mm^2^ in the adult vs. 28 and 0.12 cells/mm^2^ in the juvenile, respectively. Thus, the density of *V1R*-expressing cells was approximately 45-fold higher and 230-fold higher in the lamellae than in the recesses in the adult and the juvenile, respectively (Table 5).

**Table 3:** Density of cells labeled by single probes for *V1Rs* in the lamellae of *L. paradoxa*

		*L. paradoxa* #1		
Probes	Target V1R gene(s)	Number of labeled cells	Area of lamellae (mm^2^)	Density (cells/mm^2^)
LP02	*L. paradoxa V1R59/L. paradoxa V1R60*	36	10.78	3.34
LP03	*L. paradoxa V1R64*	26	11.17	2.33
LP04	*L. paradoxa V1R65*	16	10.32	1.55
LP05	*L. paradoxa V1R70*	13	10.86	1.20
LP06	*L. paradoxa V1R80*	9	10.45	0.86
LP07	*L. paradoxa V1R89*	3	10.51	0.29
LP08	*L. paradoxa V1R92/L. paradoxa V1R93*	25	10.66	2.35
LP09	*L. paradoxa V1R99*	20	10.95	1.83
LP10	*L. paradoxa V1R103/L. paradoxa V1R104*	8	10.79	0.74
LP11	*L. paradoxa V1R120/L. paradoxa V1R121*	7	9.07	0.77
LP12	*L. paradoxa V1R127*	29	10.63	2.73
LP13	*L. paradoxa V1R130*	7	30.15	0.23
LP14	*L. paradoxa V1R139*	3	9.27	0.32
LP15	*L. paradoxa V1R142*	8	9.57	0.84
LP16	*L. paradoxa V1R172*	7	9.39	0.75
			Total	20.11

**Table 4:** Density of cells labeled by single probes for *V1Rs* in the lamellae of *P. aethiopicus*

		*P. aethiopicus*#1		
Probes	Target V1R gene	Number of labeled cells	Area of lamellae (mm^2^)	Density (cells/mm^2^)
Paeth02	*P. aethiopicusV1R52*	18	5.97	3.02
Paeth03	*P. aethiopicusV1R53*	31	5.97	5.19
Paeth04	*P. aethiopicusV1R55*	25	5.82	4.30
Paeth05	*P. aethiopicusV1R69*	16	5.90	2.71
Paeth06	*P. aethiopicusV1R71*	6	6.29	0.95
Paeth07	*P. aethiopicusV1R83*	20	6.04	3.31
Paeth08	*P. aethiopicusV1R94*	3	6.05	0.50
Paeth09	*P. aethiopicusV1R103*	8	5.95	1.34
Paeth10	*P. aethiopicusV1R111*	4	5.82	0.69
Paeth11	*P. aethiopicusV1R116*	6	5.76	1.04
Paeth12	*P. aethiopicusV1R119*	5	5.95	0.84
Paeth13	*P. aethiopicusV1R136*	1	6.04	0.17
Paeth14	*P. aethiopicusV1R140*	3	5.90	0.51
Paeth15	*P. aethiopicusV1R141*	11	4.88	2.25
Paeth16	*P. aethiopicusV1R144*	8	5.55	1.44
Paeth17	*P. aethiopicusV1R148*	10	5.96	1.68
Paeth18	*P. aethiopicusV1R159*	12	6.22	1.93
Paeth19	*P. aethiopicusV1R160*	7	6.55	1.07
Paeth20	*P. aethiopicusV1R166*	5	6.56	0.76
Paeth21	*P. aethiopicusV1R198*	2	6.31	0.32
Paeth22	*P. aethiopicusV1R208*	9	6.21	1.45
Paeth23	*P. aethiopicusV1R213*	8	5.84	1.37
Paeth24	*P. aethiopicusV1R218*	0	5.39	0.00
Paeth25	*P. aethiopicusV1R227*	3	5.53	0.54
Paeth26	*P. aethiopicusV1R257*	1	5.15	0.19
			Total	37.57

**Table 5:** Density of cells labeled by mixed probes for *V1Rs*

		Number of labeled cells	Area (mm^2^)	Density (cells/mm^2^)	Lamella/Resess ratio of *V1R* density
*P. aethiopicus *#1	Lamellae	1168	32.76	35.65	47.66
	Recesses	9	12.03	0.75	
*P. aethiopicus *#3	Lamellae	874	14.40	60.71	670.93
	Recesses	1	11.05	0.09	
*L. paradoxa *#1	Lamellae	764	51.13	14.94	45.27
	Recesses	6	18.16	0.33	
*L. paradoxa *#3	Lamellae	1352	49.06	27.56	229.67
	Recesses	1	8.49	0.12	

**Fig. 7:**
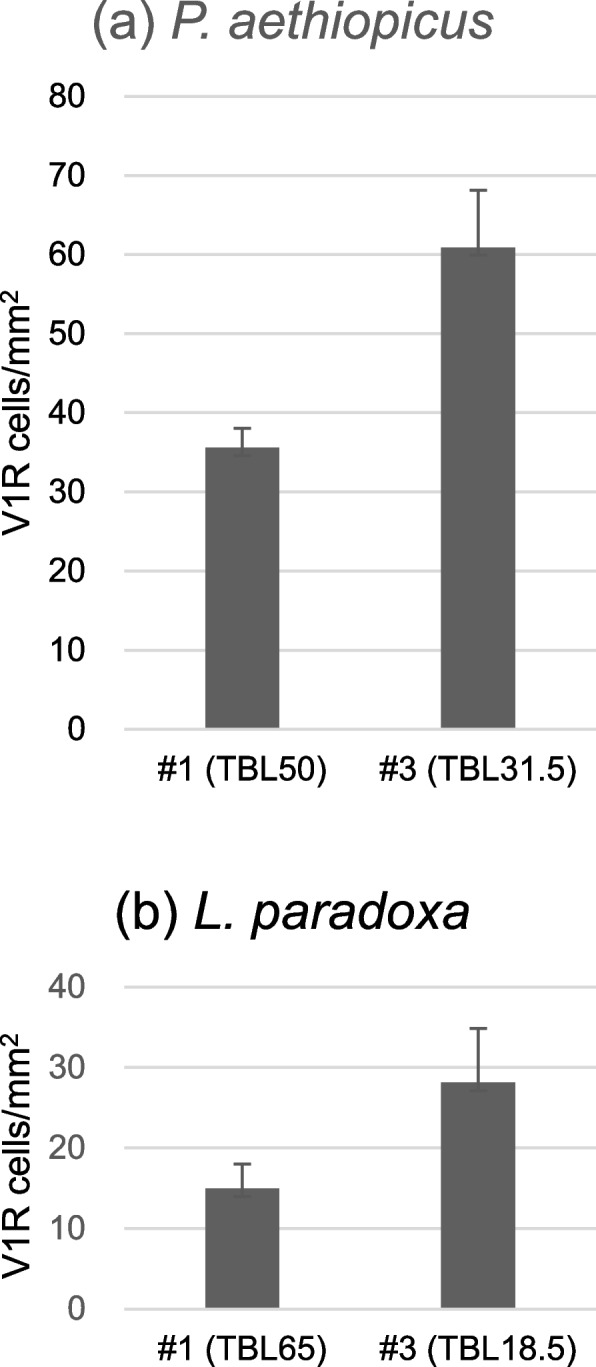
Comparison of *V1R*-expressing cell density in the lamellae between adult (#1) and juvenile (#3) of *P. aethiopicus* (**a**) and *L. paradoxa* (**b**). Data are shown as mean ± SD (*n* = 5–33, see Table 6). In both lungfish, the density of *V1R*-expressing cells was higher in the juvenile than adult lamellae

In addition, the density of *V1R*-expressing cells was higher in juvenile than adult lamellae (36 *vs*. 61 cells/mm^2^ in *P. aethiopicus* and 15 vs. 28 cells/mm^2^ in *L. paradoxa*) (Table 6, Fig. 7).

**Table 6:** Comparison of *V1R*-expressing cell density in the lamellae between adult and juvenile

		Total body length (cm)	Number of sections analyzed	Area (mm^2^)^a^	Number of cells expressing *V1Rs*^a^	*V1R* density in lamellae (cells/mm^2^)^a^
*P. aethiopicus *#1	adult	50.0	5	6.55 ± 0.25	233.60 ± 23.27	35.6 ± 2.45
*P. aethiopicus *#3	juvenile	31.5	5	2.88 ± 0.10	174.8 ± 14.97	60.9 ± 7.22
*L. paradoxa *#1	adult	65.0	6	8.52 ± 0.54	127.33 ± 25.50	14.98 ± 3.04
*L. paradoxa *#3	juvenile	18.5	33	1.49 ± 0.44	40.97 ± 13.29	28.13 ± 6.72

^a^Values represent mean ± SD

## Discussion

By examining *V1R* expression in the olfactory organ of two species of African lungfish, *P. annectens* and *P. amphibius,* we recently reported that the density of *V1R*-expressing cells was higher in the lamellae than recesses [28]. However, it was unclear whether these characteristics are shared by other species of lungfish. In addition, all individuals analyzed in our previous study had a body length of approximately 30 cm; therefore, we could not determine the relationship between *V1R*-expressing cell density and individual growth stage. In the current study, we investigated the density of *V1R*-expressing cells in the olfactory organ of juvenile and adult African lungfish *P. aethiopicus* and South American lungfish *L. paradoxa*. The results indicate that the density of *V1R*-expressing cells is higher in the lamellae than recesses in *P. aethiopicus* and *L. paradoxa*, as in the two species of African lungfish in our previous study (*P. annectens* and *P. amphibius*), and that this tendency was more remarkable in juveniles than in adults. However, the sexes of juveniles and adults were not matched in the present study. The effect of sex on *V1R* expression may need to be considered. In our previous study, we found no difference in *V1R*-expressing cell density in the lamellae and recesses between male and female *P. amphibius* [28], suggesting that a sex difference did not affect *V1R* expression at least in *P. amphibius*. The relationship between sex and *V1R* expression in other lungfish species remains unknown. It is necessary to compare the *V1R* expression levels in the lamellae and the recesses between juveniles and adults of the same sex, and between males and females of the same size.

Our intraspecific analysis revealed differences in the density of *V1R*-expressing cells in the lamellae between adults and juveniles. This evidence suggests that the density of *V1R*-expressing cells in the lamellae decreases as the individual grows. Unlike what was seen in lamellae, the density of *V1R*-expressing cells in the recesses was higher in adults than in juveniles (0.75 vs. 0.09 cells/mm^2^ in *P. aethiopicus* and 0.33 vs. 0.12 cells/mm^2^ in *L. paradoxa*, Table 5). This evidence suggests that the density of *V1R*-expressing cells in the recesses increases as the individual grows. Thus, adult lungfish showed a lower and higher density of *V1R*-expressing cells in the lamellae and recesses, respectively, compared with juveniles, and thus the abundance of *V1R*-expressing cells in the lamellae relative to that in the recesses was more than fivefold greater in juveniles than adults (48:1 and 670:1 in *P. aethiopicus* and 45:1 and 230:1 in *L. paradoxa*).

In our previous study, we found a difference between *P. annectens* and *P. amphibius* in the density of *V1R*-expressing cells in the recesses (2.4 vs. 0.1 cells/mm^2^) [28]. However, as shown in the present study, it may be necessary to take into account the individual growth stage to evaluate the density of *V1R*-expressing cells. Therefore, in the future, *V1R* expression should be analyzed in the juveniles and adults of *P. annectens* and *P. amphibius*.

Because of the small percentage of *V1R*-expressing cells in the ORCs in the recesses, the involvement of *V1Rs* in the overall function of recesses may be negligible. On the other hand, Gαo expression indicated that the RecE consists largely of *V2R*-expressing cells except for a few *V1R*-expressing cells, suggesting that *V2Rs* are primarily relevant to the olfactory functions of recesses in both juveniles and adults. A future study on *V2R* expression is needed to clarify the functions of recesses.

By contrast, the density of *V1R*-expressing cells was 50–700-fold higher in the lamellae than recesses. Most V1R genes of lungfish are classified as the tetrapod type [28, 36]. In general, tetrapod V1Rs detect volatile molecules [10]. The lamellar OE is supposed to contact the air when a lungfish moves its snout out of water for air-breathing. Thus, it is highly likely that lungfish detect volatile molecules (airborne odorants) via V1Rs.

Olfaction plays an important role in obtaining external information related to predators, feeding, reproduction, and other processes [14, 37]. The higher density of *V1R*-expressing cells in the lamellae of juveniles than adults demonstrated here suggests that juveniles are highly dependent on the V1R-mediated olfactory pathway in lamellae compared with adults. Juvenile *L. paradoxa* breathe more frequently than adults; i.e., the interval between breaths is more than twice as long in adults than juveniles [30]. Thus, the lamellar OE likely comes in contact with air more often in juveniles than adults. Juvenile *P. aethiopicus* are more threatened by terrestrial predators than are adults because juveniles live in shallow water close to shore, whereas adults live in deep water [30]. Juveniles have a wider range of feeding habits than do adults [30]. Our present results imply that differences in lifestyle, including habitats, feeding, and reproductive status are related to the differences in *V1R*-expressing cell densities between juvenile and adult lungfish.

We report changes in the density of *V1R*-expressing cells in the lamellar OE with individual growth stage. The expression of signal transduction molecules suggests that the lamellar OE contains *V2R*-, *OR*-, and *TAAR*-expressing cells, in addition to *V1R*-expressing cells [28]. Therefore, it is necessary to clarify the expression of *V2Rs*, *ORs*, and *TAARs* in addition to *V1Rs* to understand the functions of lamellar OE. Changes in olfactory function during growth would be revealed by comparing the expression of each olfactory receptor between juveniles and adults.

## Conclusion

We compared the expression of *V1Rs* in olfactory organs between juvenile and adult African lungfish *Protopterus aethiopicus* and South American lungfish *Lepidosiren paradoxa*. The density of *V1R*-expressing cells was higher in the lamellae than in the recesses in all specimens evaluated, as in the other two species of African lungfish (*P. annectens* and *P. amphibius*). This tendency was more pronounced in juveniles than adults. In addition, the juveniles had a higher density of *V1R*-expressing cells in the lamellae than adults. These results imply that differences in lifestyle factors, including habitat, feeding, and reproductive status are related to differences in *V1R*-expressing cell density between juvenile and adult lungfish.

## Supplementary Information

**Additional file 1: Supplementary Fig. S1-S5. ***V1R* expression in the olfactory organs of *P. aethiopicus *(Figs. S1-S3) and *L. paradoxa *(Figs. S4-S5).

**Additional file 2:** **Supplementary Data S1.** Nucleotide sequences of *P. aethiopicus* *V1Rs*.

**Additional file 3:** **Supplementary Data S2.** Nucleotide sequences of *L. paradoxa V1Rs*.

## Data Availability

All data generated or analyzed during this study are included in this published article and its supplementary information files.

## Abbreviations

OE: Olfactory epithelium
VNO: Vomeronasal organ
ORC: Olfactory receptor cell
OR: Odorant receptor
TAAR: Trace amine-associated receptor
V1R: Type 1 vomeronasal receptor
V2R: Type 2 vomeronasal receptor
RecE: Recess epithelium
BL: Body length
PB: Phosphate buffer
ISH: In situ hybridization
diceCT: Diffusible iodine-based contrast-enhanced computed tomography
SEM: Scanning electron microscopy
RT: Room temperature
NCAM: Neural cell adhesion molecules
DIG: Digoxigenin
SSC: Saline-sodium citrate
GE: Glandular epithelium
