# Supplementary material for: Type 1 vomeronasal receptor expression in juvenile and adult lungfish olfactory organ
Source: Zoological Lett. 2023 Mar 10;9:6. doi: 10.1186/s40851-023-00202-z (PMC9999545; doi:10.1186/s40851-023-00202-z)
Supplement: Supplementary file 1 — Additional file 1: Supplementary Fig. S1-S5. V1R expression in the olfactory organs of P. aethiopicus (Figs. S1-S3) and L. paradoxa (Figs. S4-S5). [file 40851_2023_202_MOESM1_ESM.zip › Additional File 1_ Supplementary FigS1 230213_ESM.pptx]

## Slide 1
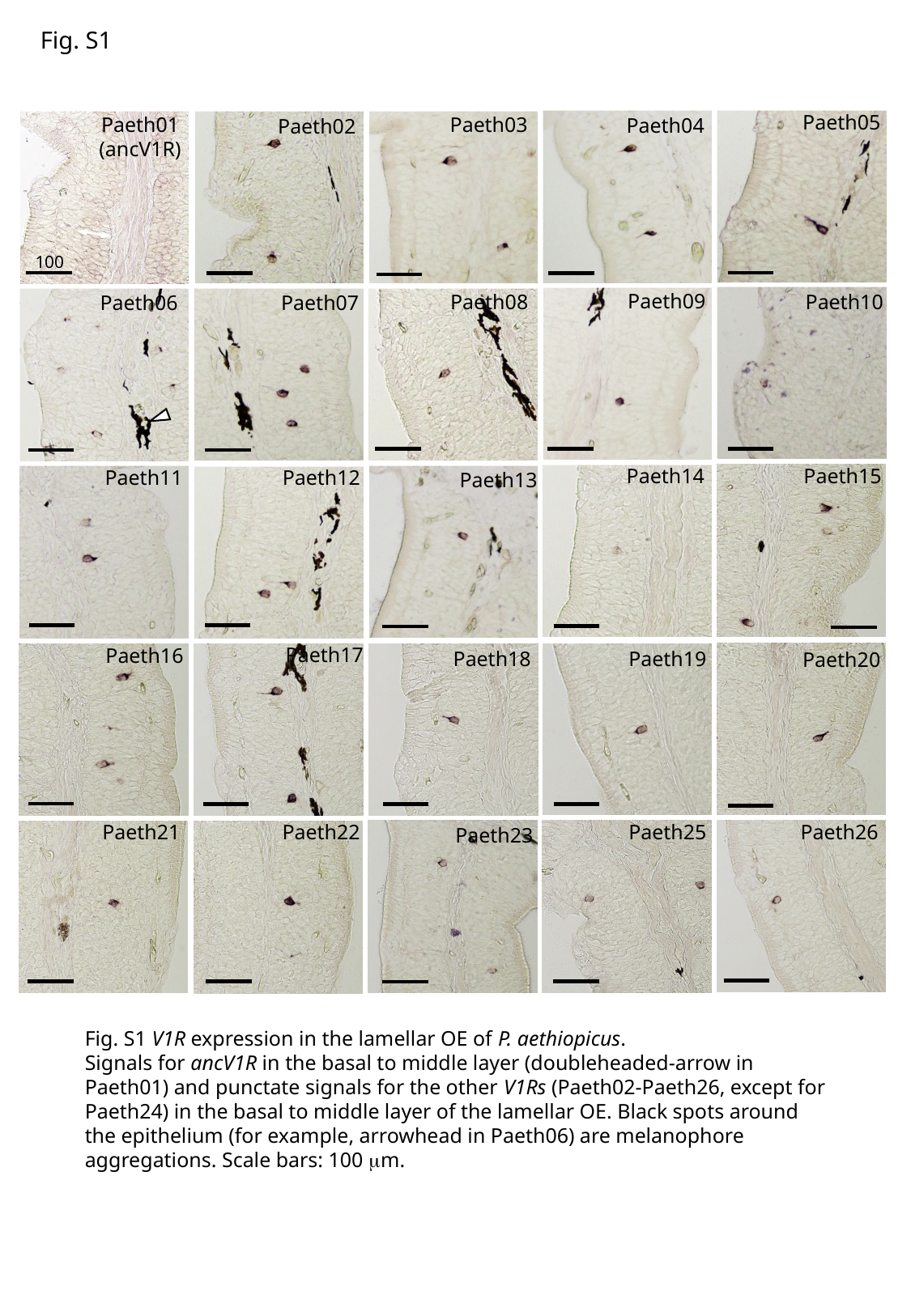

Fig. S1
Paeth05
Paeth01 (ancV1R)
Paeth03
Paeth04
Paeth02
100
Paeth09
Paeth08
Paeth10
Paeth07
Paeth06
Paeth15
Paeth14
Paeth12
Paeth11
Paeth13
Paeth17
Paeth16
Paeth19
Paeth18
Paeth20
Paeth26
Paeth21
Paeth25
Paeth22
Paeth23
Fig. S1 V1R expression in the lamellar OE of P. aethiopicus.
Signals for ancV1R in the basal to middle layer (doubleheaded-arrow in Paeth01) and punctate signals for the other V1Rs (Paeth02-Paeth26, except for Paeth24) in the basal to middle layer of the lamellar OE. Black spots around the epithelium (for example, arrowhead in Paeth06) are melanophore aggregations. Scale bars: 100 mm.
