# Supplementary material for: Type 1 vomeronasal receptor expression in juvenile and adult lungfish olfactory organ
Source: Zoological Lett. 2023 Mar 10;9:6. doi: 10.1186/s40851-023-00202-z (PMC9999545; doi:10.1186/s40851-023-00202-z)
Supplement: Supplementary file 1 — Additional file 1: Supplementary Fig. S1-S5. V1R expression in the olfactory organs of P. aethiopicus (Figs. S1-S3) and L. paradoxa (Figs. S4-S5). [file 40851_2023_202_MOESM1_ESM.zip › Additional File 1_ Supplementary FigS2 230213_ESM.pptx]

## Slide 1
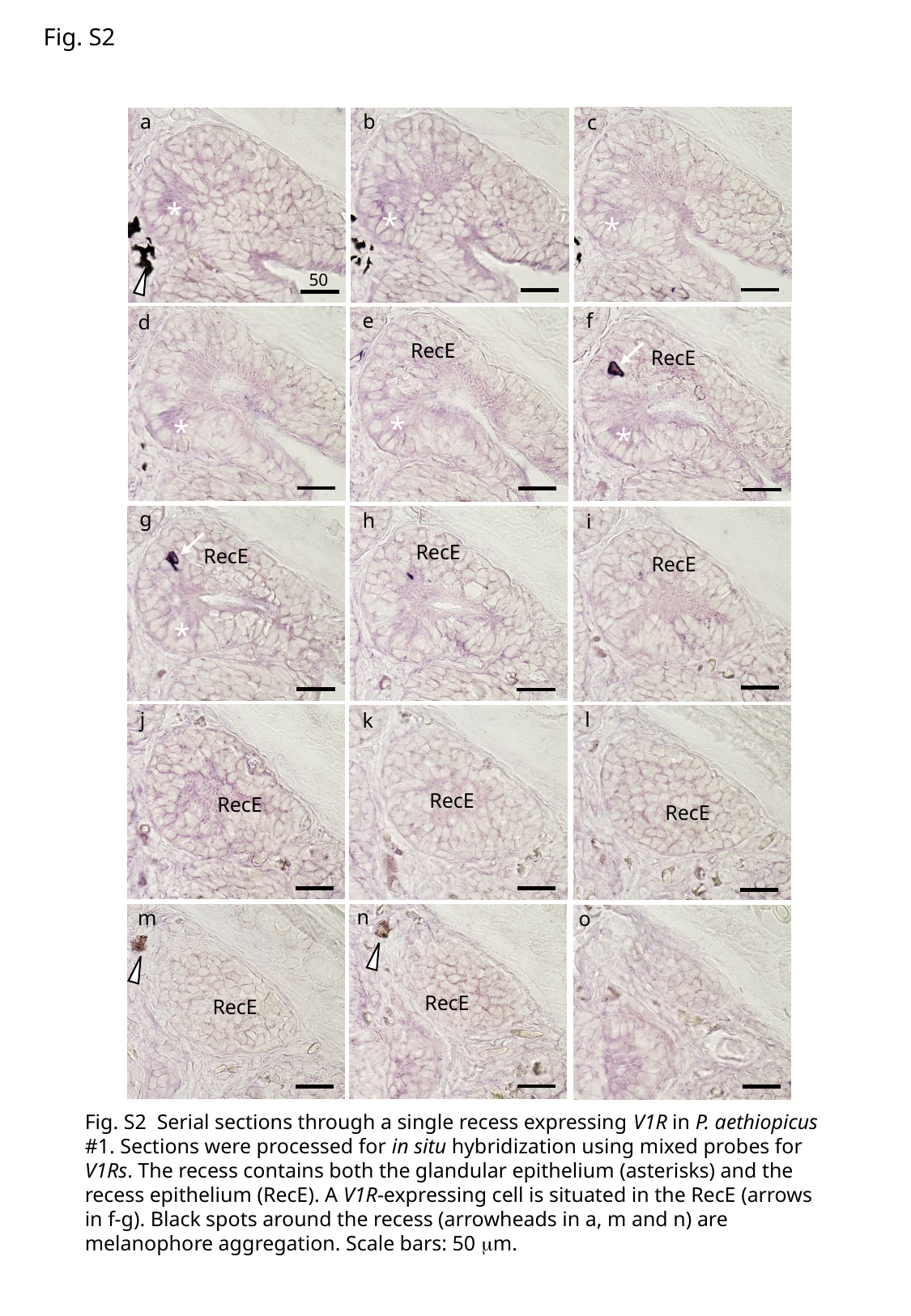

Fig. S2
a
b
c
*
*
*
50
e
f
d
RecE
RecE
*
*
*
g
h
i
RecE
RecE
RecE
*
j
l
k
RecE
RecE
RecE
n
m
o
RecE
RecE
Fig. S2 Serial sections through a single recess expressing V1R in P. aethiopicus #1. Sections were processed for in situ hybridization using mixed probes for V1Rs. The recess contains both the glandular epithelium (asterisks) and the recess epithelium (RecE). A V1R-expressing cell is situated in the RecE (arrows in f-g). Black spots around the recess (arrowheads in a, m and n) are melanophore aggregation. Scale bars: 50 mm.
