# Supplementary material for: Type 1 vomeronasal receptor expression in juvenile and adult lungfish olfactory organ
Source: Zoological Lett. 2023 Mar 10;9:6. doi: 10.1186/s40851-023-00202-z (PMC9999545; doi:10.1186/s40851-023-00202-z)
Supplement: Supplementary file 1 — Additional file 1: Supplementary Fig. S1-S5. V1R expression in the olfactory organs of P. aethiopicus (Figs. S1-S3) and L. paradoxa (Figs. S4-S5). [file 40851_2023_202_MOESM1_ESM.zip › Additional File 1_ Supplementary FigS3 230213_ESM.pptx]

## Slide 1
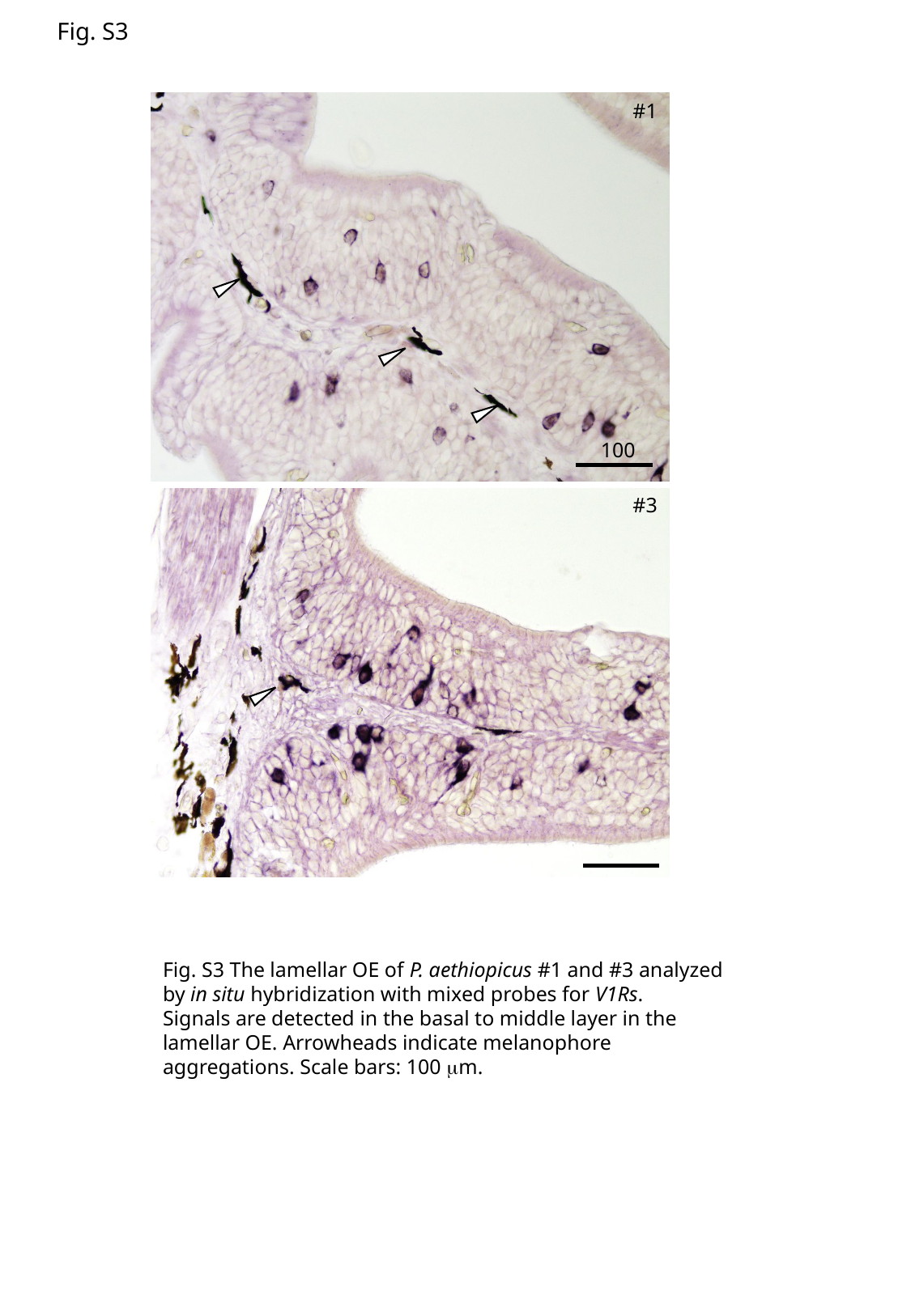

Fig. S3
#1
100
#3
c2
Fig. S3 The lamellar OE of P. aethiopicus #1 and #3 analyzed by in situ hybridization with mixed probes for V1Rs.
Signals are detected in the basal to middle layer in the lamellar OE. Arrowheads indicate melanophore aggregations. Scale bars: 100 mm.
