# Supplementary material for: Type 1 vomeronasal receptor expression in juvenile and adult lungfish olfactory organ
Source: Zoological Lett. 2023 Mar 10;9:6. doi: 10.1186/s40851-023-00202-z (PMC9999545; doi:10.1186/s40851-023-00202-z)
Supplement: Supplementary file 2 — Additional file 2: Supplementary Data S1. Nucleotide sequences of P. aethiopicus V1Rs. [file 40851_2023_202_MOESM2_ESM.docx]

>*P. aethiopicus V1R23* (*ancV1R*)

ATGGACCTGCCATCCACAGTCCTTGAAGGGATCATCTTCATTGTGTTATCTGTGATTGGCTTGCTTGGCAATGGAATACTAATTCATTTCACACTGAAAAGCTTTGTGGGACGCGTGCGAGCATCATGTGCAGTGCTTTTCTGCCTTGGCGTTGTTCACATGCTCAGGATTTTGGTGGTGAACATACTGAGTGTGATTTACAGTGTTGGTGGTGTAAGGATATTTGATTCTGCTGGGTGCAAGATTTTCAAGTTTACCTCTGCTGTCACAACCACTATCTCAATCTGGCTAACCCTCTACTTGGCTTCATTTTATTACTTGAAACTCAGTCATGTAGTCCACCCTCTAAGTGTAACACCCACAGTTAGCTGGCGTAACAGACATCTGCTGGGACTCTTTTCTCTTTGTGTAGCTGGCCTTGCAGTCTATATTCCTATTTTAGTTTATAGTGAAAAAGTCAATGATTCATTCCTGGCAAACATTACCAGTAGTCAGAAGCACGCCAGTTTGGTCTATGCTGCCTGCCAGGTTAGGTATACCAGTCACAAGCTAGAGCTTATCTATGGAACTGTGCTGTTGCTTTCCTTTGATTTGGTTCCACTAGCAGATTTTGTTATTATTAGCATTCGAATAGCACTGCTGTTGTGGCAACATCACAAAGCCAGCTATGGAGACATCTGGATTGGAGCTGACAAGACAGAGACAGAAGTTTTCAGAGCAAGTAAACTTTCAGTACTTCTAATGGGTCTTGCAGCTTGCCTGTGGATTTCTCATTTTATCTTGTATCACTACCAATCTGAGCTGAGCTCCTGCTATTTTATTCCTGCCATACTCGCAGTACTGTCCTGTAGCTATTCTTCATTAAGTCCTTACATACTCATTATAGTAAATTACAAGGTGAGGGAAAAGCTGAAAGACCTCTGCTGTTGGCATTCCATGAGGCATGCCAAAACCCAAGCTGTTACTGTTTCAGCTTCTCCATATTCAGAATGA

>*P. aethiopicus V1R52*

ATGCAGGAAATGGAATCCCACCAGCTGATAAAAGGTATAATATTCTTTCTTACAACAATCATTGGTCTCAGTGGGAATGTTCTGATTCTGGCTTTCATGGTTCATATTGCCTACCAGGAGGGAAAGATGATGTCAGCAGAAAAAATTATGACCAACCTCGCAGGATCAAACCTCATACATCTATTAACATTGGGACTTCCCCGTTCCTTATTTGTTTTAGGAATAACATATTTTTATACTGATCTTGACTGCAAAATTGATATATTCTTATTAAGAGTTTCTAGGGTCATGATCATTGGTTTGACCTGCCTGCTCAGTTGTTTTCAGTGTGTAACTCTGGCTTCCTTAAACCCACAGTGGGCAACATTAAAACTCAAGATGCAAAAATATCTAATCCTTTTAATCCTAATTGTCTGTTTAATAAGCTTGGCATCCAGTATAAATTCAGCCCTATATCCAGTTACAGGAAGGAATGCAACAAATTTAAAATACACTTTTAATCTGGGATATTGCCTTTCAATTTATCCAGATAAGCTTGTATTTGAATTGACTGGTTTTATTTCATTTGCTCGTGATGTAGCATTTGTTGTTCTCATGGCTCTTGCTAGTGCCCACATTCTTCTAATTCTACACAGACATGGAAAGCAGATGAAAGGAAAGAGGAGTTCAGATCGAAGTCAGGAAGCTGGAGCAGAGCAGCAGGCATCCAAGATGGTGGTAGCTTTTGTAGTAACATATGTGTCCCTGTTTGGAATAGAAAATACCATTTGGTTTTATCAGATCTCCGTTTCAAAGCAAGGTGATCCTTCCTATAATGATTCCCGTTATTTCTTATCATTTTGTTTTTCCTCACTTTTTCCAATAATTCTAACTGCTTATAATCAAAAACTGAGACACAGAATAATACGCTACAAATGCTAA

>*P. aethiopicus V1R53*

ATGAATGCACAAGAAATTAAAGGAGTTATCTTCTTTCTAACTACAGTCATAGGCATCTTCGGAAATGCAATCATTCTTGTGTTTATGATATACATTGCTCTTCAAGAGAAAAAAATGATGCACTCTGAGGTAATACTGTCTAGCCTAGCATGCTCAAACCTTATCCATCTTTTAACTGTAGGACTTCCACATTCTCTTTACCCATTTGGCATAATATATTTTTACACAGACCTTGACTGTAAATCTGATGTGTATTTAATGAGGGTATCTAGAGTTATGATCATTGGTTTAACCTGCTTGCTCAGTTGTTTTCAGTGTGTAACTCTGGCTTCCTTAAACCCACAGTGGGCAACATTAAAACTCAAGATGCAAAAATATCTAATCCTTTTAATCCTAATTGTCTGTTTAATAAGCTTGGCATCCAGTATAAATTCAGCCCTATATCCAGTTACAGGAAGGAATGCAACAAATTTAAAATACACTTTTAATCTGGGATATTGCCTTTCAATTTATCCAGATAAGCTTGTATTTGAATTGACTGGTTTTATTTCATTTGCTCGTGATGTAGCATTTGTTGTTCTCATGGCTCTTGCTAGTGCCCACATTCTTCTAATTCTACACAGACATGGAAAGCAGATGAAAGGAAAGAGGAGTTCAGATCGAAGTCAGGAAGCTGGAGCAGAGCAGCAGGCATCCAAGATGGTGGTAGCTTTTGTAGTAACATATGTGTCCCTGTTTGGAATAGAAAATACCATTTGGTTTTATCAGATCTCCGTTTCAAAGCAAGGTGATCCTTCCTATAATGATTCCCGTTATTTCTTATCATTTTGTTTTTCCTCACTTTTTCCAATAATTCTAACTGCTTATAATCAAAAACTGAGACACAGAATAATACGCTACAAATGCTAA

>*P. aethiopicus* *V1R55*

ATGAAAATTGAAACACGTCATATCATAAAAGGAGTTATCTTTGGAATAACTACTGCTGTTGGCCTTGCAAGTAATATTGTGATTATGTTAGCCCTATCTAGCATTGCTTACAAGGAGAAAAAGCTGATGCCTTCTGAGACAATACTTTTTACTTTGACAGGAGCAAACCTGCTGCTTCTTTTCAGCCTTGGGCTCCCTCACTCTCTTTTTGCATTCGGAGTGAAATATTTCTACACAGATCTTGACTGTAAATTTGATGGACATTTCTTACGATCATCTCGGGTAATGATCATTGGCCTAACCTGTTTACTGAGCTGCTTCCAGGGAGTAACACTGGCTTCTTCTAATCCCACCTGGGCTGTTATTAAAGCCAAGATGCAAAAATATCTTTCCCTTCTAATAGCATTTATTATAATTCTTAGCACAGTGTCTAGTTTAAGTTCAGCTTTATATCCCATTGTTAGTAGCAACAGTACAGATTTGAAACATTCTATACATTTGGGGTACTGTATTTCAATGTATCCTAATGCCTTTTTATTCATGCTAATTGGAATGATTTCCTTTGCTAGAGATGTGTTCTTTGTAGTCCTTATGGCTGTGGCAAGTACTTACATACTTCTAACTTTGCACAGACATGGAAAACAAATGAAAGGGAAGAGGAGCTCTGATCGAAACCAGGAAAACTCCGCTGAAGAACAAGCGGCTAAAATGGTTGTTATTTTTGTTACCATTTATGTATTACTGTTTGGAATAGAAAACATAATTTGGTTTTACCAGATTTCTGGTTCTGATGAAGGCAACACAGCTTTAACTGATACTCGCTTTTATCTTGAATTTTGTTTCTCCACTGCTTTTCCTTTTGTATTAATTACATTCAACAAGAAAGTTAAGTGTCAGCTCTCATGTTCACTTACTTAG

>*P. aethiopicus* *V1R69*

ATGGAAACTCAACACATCATTAAAGGGACTGTTTTCTGCCTGACAACGTTCATCGGACTTTGTGGTAACATTTTTATTTTGACCCCATTGTCACACATTGCTTACCAAGAAAAGAAGACTCTTCCATCAGAATTAATTCTCATTATGCTAGCTGTGACTCACATTGTACTTTTAATAACATTAGCTCTTCCTCAATCCCTTTACGAGTTTGGAATTAAGTATTTCTATACAGACCTTGAATGCAAACTGGATGCCTACTTCAGTAGAGTTTCCAGAGTGATGTCTATTGGACTAACCTGTCTGCTAAGCTGCTTCCAGTGTGTAACTTTGAATTCTGCTAATCCAGTTTGGGGACTCTTCAAAGCATGGATTCAAAAAAATGTCATAGTTATCATTTTGGGTATATGTGTGCTCAGCATGGTCTCCAGTATTAGTTCAGCCCTTTATCCTGTAGTTAACACAAACCTTACTGATCCAAGGCCAATGTTCAAAATGGGATACTTCTATGTTGTTTTTCCTAACATATTTCTGTATCAGCTTACTGGTTTTATCATGCTTAGTAGAGATTTAGTATTTATGACATTAATGGCTTTAGCTAGTATGTATATATTGCTGGTTTTGTACCGACATAATGAACAGATGAAGGGAATGCGGAGTACTGACCAAACAAATACAGCTGAGCGGCAGGCAGCTAAGATGGTTGTAACGTTTGTTGTATTGTATGTTCTGTTTTTTGGCATAGACACCTCCCTTTTGTTTTACCAAATTTCCATCTCAAATGAAGGTCCTGCTGCTATAAATGATGTTCGCCAGTTCTTAGCATTTTGTTTTCCATCTGCCTTTCCATTGATACTCCTTGCATTCAATCAGAAAGTTAGGTATGCAGTGACTTGCCACTCTGAATCATTGTGCAGTTAA

>*P. aethiopicus V1R71*

ATGGATTCAGAAAGCATTATCAAAGGCTTTGTCTTTGGTTTAATAACTTCCTTTGGGACTTTTGGCAATGTGATTGTTTTGGCTACTCTGGTCTGGATTGCCTGCCAGGACAAACTTTTACCCTTTGAAGTTATACTCTGTAATCTGGCACTATCAAACTTTGTCTTTCTTCTGACTGGGGGTGTTCCTGACATGTTATTTAAATTCAAGATCAAATATTTCACAAAAGATGCTGATTGCAAGGCTGATATTTATATTGCAAGGGTTTTTCGAACCATGACTATTAAATTAACTTGTCTGCTGAGTTGTTACCAGTATTCAGTTATCACATCACCATCTTCTAAGTTGTTCTTTTTAAAAGTTATAATGAAAAAATATTTTGTGTTAATTATGATATTCCTCCTCATCTTAAGTGTTTCATCAAATGTACCTGGAGCTCTGTACCCTGTGGTTAATGCTAATACTAGCAATTTGACATATGCATTCAATTTAGGAACTTGCACTGTAATTTATCCAAATGGTTTTATATTAAATATGACAGGCTTCTTGAATTTTACTCAAGATCTAATTTTTATGACCCTCATGGCTGTTACAAGCACCTGCATTTTAACAATTCTGTATCGACATGGGAGACAGATGAAAAACATACGACATTCCAAAGAAAACCAAGAGACTTCAGCTGAACATCGAGCATCTAAAGCAACTGTAACTTTAGTGATTTTATATGAAATTTTGTATGGAATAGAATGTGCAGTTTGGTTTTATCAGTTATCAGCCTCAACAAATCACCTGATCTTAAATGATGTGCGGCATTTTCTGTCCTTGGCGTTCCCATCTTTTTTTCCAGAAATTATACTCTTTTTTAACCAAAAGCTGAGAAAGAGATTGCATTGCTGA

>*P. aethiopicus V1R83*

ATGAATGATGCAGATATTAGGGCAAGCCTCTCATGGCAAACACAGCAGTGGAGACTCATAATTGTGTGTATCAGGACTCTTTGGGGACTGAAGACAACCAATGTCACACTTGTCAGACCAGTTTCAAGTTCTACTCACAAAGTGACATATCTGACAGACCTAAGAACTGAGTTCTTCGAGTCAATACTTGCCAACCCACCAAGAATGAGCTTGTACGAGCACATAAAAGGAGCTGCACTTTTTGCAATGACTTTGTTTGGATCGTTTGGCAACATAGCAACAATTCTGTCATTTATTCAGATTGCATATCAGGAAAGAAAGCTTCTACCTGTTGAAATAATTCTGTCATGCCTTTCAGGAGTAAATCTTTTGATATTACTCTCACGAGGAATACCATATCCTCTGTTCATCTTTGGTGTGATTGTATCATTTAATGACCCAGCCTGCAAAGCTGTTTCTTACATACACATATGGTTTCGATCACTGGGTGTCAATTTAACTTGTCTTCTTAGTTGTTTTCAATGTATAACTATTAGCTCTGGTTCTGCAAAGTGGGTCAAACTAAAAGGAACTGTACAGAACCATCTTTTAAGTCTTATAAGTTTTCTGTGCTTAGTTAGCATGGCATCGAGTGTGGACATCATTTTGTTCGGATCTTCTGGTACTAATGTTACTGGATTACAAAATACAATTTTTAATGGATACTGCCTTAACACTTTACCTAGCAAGATTGTATTTGACACAATTAACTATGTTATTTTTGCTCGTGACGTTGCATTTCTGCTTCTTATGACATTATCCAGCTGTGTTATTTTAATAATCTTGTATAAACATCAGAAGAGGGTAATTGGCATAAGAAGTTCAGAGAAAAGCACAAAAACTACTGCAGAAGGGCAGGCATCTAAGACTGTTGCTATTATAGTTGTTATGTATGTGCTTTTCTTTGGCATGGGTACCACCATTTGGTTTTATGAAGCAGTAGCTGATACAAAGGTTAATTTCCTTGCAGTGATACCTGATTTTCTTTCTGTATGCTACTCTTCCTTCTTCCCTGTGGTTATTATTGTGTTTAATAAAAGAATTCAAAATGTACTCAAGGACTATTTTGGCAAGCATGGAACAAAACATTTTGGTGATCTTGGAGACTGA

>*P. aethiopicus* *V1R94*

ATGGATGTAAGGGCAATATTGAAAGCTACTGGTTTTATTTTGCTTCTTGTCATTGGAATACCTGCAAATCTTTCCATTCTGATGTCATTCTTTACTTCTGCAGTTTCTGAAAAGAAGCTCATGCCAACAGACTTCATTCTTACCAAACTGTCCTTTGTAAATGTTGTTGTTGTACTTGTACGAGGAATTCCACAGTCTCTTACAGCCATAGGCATGCAGAAGTTATTCAATGACTATGGGTGCAAATTTGTAATCTTTACTTACCGTGTTTGTCGAGCGATGTCTGTATGCATAACAGCTGTACTGAGTATCTATCAGTGTATTGTCCTTTTACCTCCTACAACAAAATTTATGACATTGAAACAAAAGGTGTCTCAGAATATTTTTCTTCTCTTCCTTCTTCTATGGTGTATCAACTGTATAATTTACATACCAGCTGGTTTTATGTATTCTCAAGCTGAAGTAAATTCAAGTATTCCCAAGTATGCACTTAATTTAGAGTTTTGTTTTGTTTTGTTTCCTCATGAGGTATCATACAGAGTTAATGGAATAGTTTATACCTTTCGAGACTTTTTATTTGTTGGACTTATGACACTTGCCAGCAGCTACATTGTTATAATTCTATACAAACATAATAAAAAACTCCAGAACATCAGAAGTCCTGACCAGAAGCAAAAAAGTGCCACAGAATTAAGGGCAGCTAAGGCTGTAGTTATTTTGGTTACATTGTATCTTGTGTTGTTTGGACTTGATAATGCCATCTGGATGTATACCCTTAGTGTTTCTAATGTAGCACCTGCAGTTTCTGATGCCCGTGTCTTCTTTGCCTCACTGTATACTGCAGTTAGCCCTTTGGTAATAATTGGGACAAACAGAAAAATTCAACAAAAGCTGAAATGTACTTCTGCTAACATTGAGCATAACATACGAGAAAGCACTATCACACATTTAGCTATGAGTAAGGAAGAGAAGAATTATTAA

>*P. aethiopicus* *V1R103*

ATGGGTCCTCAGGATATCCTCAAAGGAACTATCTTTTCTGCAACCACCATCATTGGGTTATGTGGTAATACAGTGGCTTTGATATTCTTGCTCCAAGTTGCACACAACGAGAAAAAAATGATGACAAATGAGGTAATACTGTGCAGCTTGGCTGTCTCAAACTTAACTCTTCTTTTCACGCTGGGACTTCCCCATTCTCTGTTTGTGTTCACAGTTAAGTATTTCCACACAGATTTGGATTGCCAAGTTGATCTCTATTTACTGAGAGTGTCCAGAGTCATGGCTATTGGGTTAACTTGTCTGCTGAGCTGTTTCCAATGTACAGTACTTGCATCTTCTTCACATTCTTGGTGGACTGCCCTCAAAGCCTGGATACAGAGGTACCTTGGATGGATCATCATCTTCCTTGTTCTTACAAGCATGGCCTCAAGCATTAATTCTGCCCTATTTCCAGTCATCAGTACAAACCTCACTAATTTGCCCTACACCTTCAACTTGGGCTACTGTTTTGTGATTTACCCAGACAAGCTATCATTTGAAATCATTGGTTTTGTTGCATTTGCTAGAGATGTGGTCTTAGTAATCCTCATGGCTTTGGCCAGCAGTTATATCTTGTTTATTTTGTTTAGGCATGAGAAGCAGATGAAAGGAAAGAGGAGTTCTGACAGAAACCAAGACAAATCTGCAGAACGTCAGGCTGCCAAGACTGTTGTCACTTTGGTTGTCCTTTATGTGTTTTTCTTTGGGATAGACAATGTTGTCTGGTTCTATCAGATCTCAAAGTCAAATGAAATCAATCATTTTGTAACAAACCTGAGACTTTTTGTGACTTTCTGCTTTCCATCACTTTTTCCATTTATTATTATTTTCTTCAACCAAAAGCTGAGAGACAAAATAACATGCTCAGTCAGTAAAAATAAAACACAGAAAATTGAGACACTGACATCTGTAAAG

>*P. aethiopicus* *V1R111*

ATGGAGAAACAAGTCATAAAAGGAGCTATCTTTAGCTTAACAACTATTATTGGAATTTCTAGTAATATATTGATTATGCTTACTCTGTCCAACATTGCTTACAAGGAGAAAACATTGATGCCTTCTGAGACCATACTTTTAGCTCTGACAGGGGCAAACCTGTTACTCCTTTTTAGCTTAGGGCTTCCTCATTCTCTTTTTGCATTTGGCATTAAATATTTTTACACTGACCTTGACTGCAAATTTGATGGACATTTCATGAGATCATCTCGGGTAATGATTATTGGTCTAACCTGTTTACTAAGTTGCTTTCAGGGAGCAACACTGGCTTCTTCTAATCCCATATGGGTTATTATTAAAGCCAAAATGCAAAAATATATTTTGCTTCTCATAGCATTTATTTTATGTCTTAGCACAGTCTCCAGTTTAAGCTCTGCACTGTATCCTGTTGTTAGCATTAATGGGACTGAATTCAAGCATGCTATAAACCTGGGTTACTGCCTTTCAGTTTATCCCAGTAAACATCTGTTTGACCTTATTGGATTTATCTCCTTTGCTAGAGATGTAACATTTGTGATCCTCATGGCCACAGCTAGCATCTATATTTTGGTGGTTTTACTTAGACACAGAAAGCAGATGAAGGGGAAAAGGAGTTCAGACCACAGTCAAGATACCACAGCAGAGCTACAAGCAGCAAAGATGGTTGTTACTTTTGTTACTTTATATGTACTGCTTTTTGGAACTGAGAACACCATTTGGTTTTATCAGATCTCTCTTGCTAAGGAAACCATTATGGCCCTTAACAACACAAGGTTTTACCTAGCTATTTACTTTCCTACGGCTTTTCCATTCATACTAATTGCCTTCAATCAGAAAGTTAAACATAAGATCAGATGCACCTAA

>*P. aethiopicus* *V1R116*

ATGACGCTATATAACTTTGCAAAGAGCCTTTCTTTTTTTCTCATAACTCTAATTGGAGCTTGTGGCAATATAATTGTAATATATTCATTCCTACAAACTGCAAAGAAGAAAATAAAACTTATGGTAGTAGAGAAAATACTGCTGAAGTTGTCGGTAGTAAACCTCATATTGCTGCTCTCGAGAGGCATTCCTGAACCATTGCTTTTGCTTGGAGTGAAAATATTTTTTTCCGATGCTGCTTGCCAACTTGTCTCCTTTGTATATCTATGGTTCAGAGGATTAGCGCTGAGTCTGACTTGTCTTTCAAGTTCTCTACAATTTCTTATAATTGGAAGCACAAATGAAAAATGGAGAAAGATAAAAAATGTCATACAAAAATATATTTTCATTATTATTGTCTTTCTGTACCTTTTAAGTATGGCATCCTGTGTAGATGCCATTTTGTTTTCTGCTTCTGTAACTAATTCAACAGATGTGGAAAATGCTATTACTAATGGTTACTGTATCAACATCTTACCATCTAAACTTGCGTTAGACTCCATAGGGTTTCTTATTTTTGCCAGAGATCTTCTGTTTGTTATTGTGATGTTTCTGTCTAGTTTGCATATATTATTAAAGCTGTTCAGACATAACAAAAGTGTAAAAGGTATGAGAAGTTCTAACAGAAACACAAAGATGACAGCAGAAGGTCAGGCAGCTAAGACTGTTGTCACATTAGTTATATTATATGTGTCAGTTTTTGGAATAGGTACAACTGTGTGGCTTTATGAAATAGTAACGCAAACAAAAATTCCTGCCACTTCTGAACTCCGTTACATCACATCCATGTGTTATGCTGCCTCCTTTCCTTTTATAATTATTGTTTTTAATAACAAAGTTAAGCATATCCTTAAAACTTGTGTTACAACAACATCAGAAAATTCTGTGGATTAA

>*P. aethiopicus* *V1R119*

ATGGATCTTTCCATTATCATCAAAGGAATTTTCTTCTTCCTTCAGACAAGTACTGGTGTTCTGGGTAATATAATAATCCTTACGTGCTATGCACATATTGCCTTCAATGAACGGAAACTGATGCCAGTTGATAGCATCACATCCCACCTTGCTGTTGTTAACATGATAGTACTACTTACAAGAGGAATTCCACAGACAATGACTTCTTTTGGATTACAGAATATTTTAAACCACCATGGCTGTGTCTTCGTCATTTTTGTTTATTCAACAGTAAGAGCTCTTTCAGTGTGTGTCACTTGCCTTTTAAGCTTGTTTCAGTCTGTTACCATTGCCCCATCAACCTGCAGTTGGGCCAGCCTCAAAATGAAAATCCCGCAATACCTTATGCCATGTTCATTCATACTCTGGGTGGCAAATATGGTTATGTGTTGTGGCCTGCTCCTTTATACAACTGTCCCTCAAAATGGAACAGTTCCAAAATATACAATCAGTACAGGGTACTGTCATGTAAAGTTCCCTAGTGAACTTGTATTTAATGTTTATGGGGCTGTATATACAACACGAGATGCTTTAATTGTGTGGGTGATGATTATGTCAAGTGGTTATATTTTGTTTACACTGTACAGACATAACAAACACGTTCGGAGTATAAGAAGCTCAAACTCAAGTAAATCTACAGCAGAGATAAAAGCTGCTGAAATTGTCATATGCTTGGTAATACTTTATGTCATGTTTTATGGGATTGATAACATAATTTGGATATACATGTTAATTAAAACATCTGCATCTTCTGAAGTTGAATATTTAAGAGCATTTTTTTCTTCTAGCTATGCATCTCTAAGTCCTTTTCTAATTATTAGTTTTAATAAGAAGATTCAACAGAACCTTTGCGTTAAAACTAGCTAA

>*P. aethiopicus* *V1R136*

TTCTTATATATTTCATATCTGGATAGCAAGCTGAAGAAACCAGAGTTGATCCTTTGCAACCTGGGAGTGGCATGCTTGCTCCTGATTCTTACACAAGGGGTTCCATATTCAATGTTTTGGTTTAGAATGGCAAATCCCTTCAGTGATGCTGCATGTAAGGTTATTGTCTTTACATTTAGAGCTTCAAGGTGCTTGTCTATTGTTCTGACATGTCTTCTTAGTTGCTTTCAGTGTGTGACCATTTTCACTAGTGTTAACTGGGTTAGAATCAAACTCAAAATGAAAAATTATCTCAAAGTAATTATTGCTTTGCTTTACATCTTCAGCGCATTATTCAGTGTCAATCTACCATTTTCAACTATTTCTAGTTTCAACATCACAGCCACAGACTATGCAACAAATTTAGGATATTGCATTGTGATCGATCCAAGCACACTACTGTTTGAAGCCATAGGATACAGTGTTCTTACTCGTGACACGATATTTGTGGTTCTTATGGCCATGGCCAGCAGCAACATTTTGTTAATATTATATAGACATGGAAAGCAGGTAAAAGGCATAAGAAGCTCAGACCACAGTAAGGAGAGTTCAGTGGAGAGTAAAGCAGCGAAGACAGTAGTAACTCTAGTTGTGCTCTATGTTTCCATTTTTGGAATAGACAGTACGATCTGGTTATATCAGATAGCAACATCGAGCAAAATTTTAGATGCCGTGTCTGACATTCGCCATTTACTTTCTGTGTGTTATGCATCTGCCTTTCCTATTGTTAGTCTAATATTTAATCCAAGAATCAGAAATGTATTTAACTGCTATGCTGAACAGTAG

>*P. aethiopicus* *V1R140*

ATGGAAGTAATTGTCATTCTGAGAGCAACAGGTTTCATGTTGTTGCTTGTCCTTGGAGTTCCAACAAACCTCATTGTCCTGGTGTCATTTCTACTCACAGGAATGTATGATAATAAACTAATGACAACAGACATTATTCTTACAAAATTATCATTTGTGAATCTTGTTGTTGTCCTTTCAAGAGCTACACCACCATTTCTTACGACCATAATCCAAAAGAAGTTATTTAATGACCTTTTTTGCCAATTTCTGATGTTTGTTTACCGTGTTTTTCGAGCGATGTCCATATGTGTAACAGCTTTTCTGAGTTGTTATCAGTGTGTTGCTCTTTTGCCACGCTCATCTACATGGGTAACTTTGAAGCAAATATTTTCCCAGAATGTGTTATCTATCTTCTTTTCCCTCTGGTGCATTAACTCTGTAATTGACATTCCTGAAGCCTTCATATATACTTGTTCTGATCTGAACTCAACCATTCCTAAATATACTCTTAACTTGGAATATTGCTTTGTAGTTTTCCCTCATAGTTTTTCATACATAGCCAATGGGATTGCTTATACTTTCAGGGATTTTTTATTTGTTGGATTTATGGCATTGGCCAGTGGTTATATTGTCACAATTCTCTATAGGCACAAAAAAAAAGTTCAGGGGATTAGACATTCTGACCATAATCAAAGAAACACTGCAGAAACAAGGGCAGCTAAAGCTGTTGTATTACTGGTGACAGTGTACATTATTTTATTTGGAATTGATAATTTTATATGGATTTACACTTTGACTTTCTCTAGTGTCATTCCTGCTATTTCCAATGCCCGTGTATTTTTTGCCCTAATGTATGCTGCTGTCAGTCCTATAATAATAATTGCAACAAACAAAAAAATTCAACTCAGACTGAAATGTATATTTCTTGGTAGTGAAAATAAAATACAAATAAATAGTACAATCACTGGGCATTTAAAAGACAAGAATCAGTATAACCGTTTTAACATGTGA

>*P. aethiopicus V1R141*

ATGATGAATACTTATGAAACTCAAAAGGCGGTGTCATTTCTTGTCCTTCTGATAGTTGGGGTTCCAGGGAATGTTCTCATTCTTGTTACCTTTGCTCAGATTACTTACTCTGATCACCACCTGCTTCCGGCTGATGTTATACTGACAAATTTAGCTTTTGTAAATCTTCTCCATGTTCTAGCCAGAGGAATGCCACAGACACTGTTTTCCTTTGGAGTCAGGAATATGTTTAACACTCTTGGATGTCAACTGATAGTTTTTATCTTTAGAGTTTCTAGAAGTCTGTCCATTTGTCTGACTTTCTTGCTGAGTGCTTTTCAAAGTGTCACAATTGCACCTGCTGATTCCAAGCTGTCTTCATTTAAACAAACATTGGCAAAAAATAACTTATATTTCATTATATTCTTTTGGCTTTTAAGTGGAACTACCAATTTTGCATTCATTTATAGCACATCACAAACAAATTTAACAGTGTACCGCTTTACTGTCAACTTGGATTATTGTTTTGTACAGTTTCCTGGAAAGGAGTCATATGAAAGCAATGGCTTTATGTACCTCATCAGAGATCTTATAATTGTTATACTCATGGCTTTATGTAGTATTTATATTCTTTTTGTTTTGTATAGGCACAGCAAAAGTATAAAAGGTATTCGAAGTTCAGATCGAACACAAAGTGTTTCAGCTGAAGCCAGGGCAGCAAAAACTGTTGTAACTCTAGTTGTGTTATATGTTATACTTTTTGGCATTGATAACATGATATGGGTTTATACCCTGCTAGTGCCCCAGAATGCAGTAGTGGCATCAGATATGAGACACTTAATTTCTTCCCTCTATGCAGCAGTTTTCCCACTTGTTATCATCTTTTTCAATAAGAAAGTTAGCAGTAAGTTACCATGTACCAATATTTGTAAGCACTCTAGATTGATTTCGACAATTCTAACACCATCTTTAATTTCTGCACTGCTTTACAAATATATCGACATATTTTTCTGTATTTATGTTTTGGCTACAAGTACAAAAGTTTACTTCTGTATTATGAAATATAAAGACTTGGGTTTATAA

>*P. aethiopicus* *V1R144*

ATGGATATCTACAATGCATTCAAGGGAATTATATTTCTATTGCTAGCAGTTACTGGCATTCTTGGAAATGTAATTATAATGTGTTCTTATGGAATTATATTTCTGAAGGAAAAACTGAAGCCATCAGACATAATTATCACTGAACTGGCAGGGACAAATCTGATTATGGTCCTAACACGGGGACTTCCACGTGCTCTGTATGGTTACGGTTTCAAAAGGCTCTTCAATGATACTGGCTGTAAATTCATTATTTTTTTTTCTCGGGTTTCTAGAGCTATGTCTTTGTGTCTAACATGTCTGCTAAGTTGCTTTCAATGTGTAACACTTGCATCTTATGATTCAAAATGGTCTTATATAAAAGTGAAAATGCAACGACTGTTACCATTAATCTTTGTTTTCCTTTTGTTAATAAATTCATCAGCTGATGTTTTGAGGACAATGTATACAGTAGCTGGTAGAAATGCCTCTAATCTTCCATTTACAAATGATTTTGGATACTGTCTTGTAATTTACACAGACAGATTATCATTGCAAGTAAATGGATTTGCCAGTTTTGCCCGTGACCTGATATTTGTTGTTCTTATGGCTTTAGCTAGTGTCAAAATGCTAATAATTTTGTACTGGCATGGAAAACAAGTAAAAAACATAAGAAGTTCTGACCAAAATCAAGAAAGTACAATGGAGAGCAGAGCTGCAAAAACTGTTTCCACTTTAGTCAGTTTGTATATTGTTTTCTTTGGGATAGACAGCACCATATGGCTGTATCAGGCCATGATGTCTGATGAGATCCATGTTGTTTTATCAGACATG

>*P. aethiopicus* *V1R148*

ATGGATTTCTATGCTCTTGTGAAAGCACTTGCATTTTTAACATTGGCATTTACTGGAATAACTGGAAATGGATTTATTATTCTATGCTTCTGTAAACGTTCTTGCAAACAACAGAAACAGAAGACAACTGATACAATCATTTCAGAGCTGTCAGTGGCAAATCTTTTGATGTTATTAACAAGAGGGTTACCAGATAGCCTTTTTGCATTTGGAATTCGGAAGCTTTTTAATGACATCGGCTGCAAATTTATTTCATTTATTTCCCGGGTATCTAGGTCTATGTCATTATGCTTAACATGTTTGCTTAGCTGTTTTCAATTTGTCACACTTACTTCATCAAATGTTACATGGGCATATGTGAAAAACAAAATGCAAGCATTTCTTGTACTTATTGTTCTTTTTCTGCTCCTTATAAATTCATGTATTTATCTTGCTGGTTCTATTTACTCAGTTTCTGGTGGCAATTACACTGACTTAAAATATTCTTCTAATCTTGGCTACTGCATTGTTATTTTTCCTAGCAAAGAATCATTTGAGGCCAGTGGTTATTGTAATTATGCTAGAGATATTATTTTTGTAGTTATTATGGCTGTAGCAAGTTCCAGAATATTATTCTTATTATACCAGCATGGGAAGCAGGTAAGAGGAATTAGACAATCTGACAAGAATCAGGAGGGTACAGCAGAGAACAGAGCAGCAAAGGCTGTCGCCACATTAGCGAGTTTGTATCTCTTCTTCTTTGGGATAGACAGTACCATTGCACTATATCAGGCATCAGTGTCACGGATACATTTTATTGTACCAGATCTGCGGACTTTCTTCTCAATGTGTTATAGTTCAGTCTTCCCGATTGTCACTGTACTTTGGAACAAAAATAGAAAAACAAAAGTAAATTCTTCTAGTGATAAAGAGAATGAAGTAAGAAATACTTCCTATATGCAGCAATAG

>*P. aethiopicus V1R159*

ATGTTTATTTCAACTATCATCAAGGGAGTTTGCTTCTTGATTCAGACAAGTACTGGAGTCTTGGGCAATTTAGTTATCCTACTAAGTTATGGACACATTGTCTTTAACAAAGGAAAACTGATACCTGTGGATGCAGTCATATTCCACCTTGCTTTTGTTAACATTATAGTTCTACTTACAAGAGGAATCCCACAGACAATGACATCTTTTGGATTACATAATATTTTAAATCATCATGGATGTGTGTTTGTCATTTTTGTTTATTCAACAGTTAGAGCTCTTTCTGTGTGTATCACTTGTCTTTTAAGTGTGTTTCAATCTGTTACTATTGCTCCAGCAACCTGCGGTTGGGCTTGCCTCAAAATGAAAATCCCACAGTACCTTATGCCATGTTCATTTATACTCTGGTTAGCCAATATGGTTATGCATTGTGGCCTGCTCTTCTATACCACTGTGCCTCAAAATGGAACAGTTCTTAACTATACCATTAATATGGGCTACTGCAATATACGACTCCCTGGAGAAGTTGCATATATTGTTTATAGTGCTATATACTCAGGGAGAGATATCATGATTGTTTCTATGATGGTGGCAGCAAGTACATATATTTTATTGCGATTGTACAAACATAATACACAAGTTAAGGGTATAAGAAGATCAAATGCAGCTCTGAAAGGTACAGCTGAGGTTAAAGCAGCTGAAACTGTCTTATCATTGGTCACACTTTATGTCACATTTTACGGAATTGATAATGTAATTTTCATTTACATAATCATTAAGATGTCTACTTCTACAGAATTTGTAGATCTACGAGTGTTCTTGTCATCTTGCTATGCAACTCTGAGTCCTTTTCTAGTTATCCATTTTAATAAGAAGATTCATAAGAACATTTGCCTTAGAACTGGAAAATAA

>*P. aethiopicus* *V1R160*

ATGGAAACATCACATCCGGCAAAAGGGATTATATTTCTCCTGACCACTGTCACTGGAATTTGCAGCAACATCCTTATTCTGATTTTTCTTTGTTCTGTAACCTATGAAGAGAAGAAGATTATGTCAACAGATATCATACTGTTGATCTTGACAGGATCCAACCTTTTGCTGTTCTTAACAATGGGGGTACCCCATTCTCTGTTCGACTTTGGAGTGAAATATTTCTATAGTGATCTTGAGTGCCAAGTTGACCTCTTTTTATTACGTGTATCGAGAGTCATGATAATAAGTTTAACATGCCTGCTCAGTTGTGTTCAGTGCATAATTCTTGCATCTTTTCATTCTAAATGGATATTTTTAAGGTTAAGAATTGAAAAATACCTAGCTCACATTATTGTATTGCTTTTTGTGTTAAGCCTGGCTTCTAGCATTAACTCAGCACTTTTTACAGTTGTTACCATTAACGCTACTGATATACAACTTACTGTAAAGTTAGGGTACTGTTTTGTGATTTACCCTAACTTACTTTTGTTTCAGTGTATTGGCTTTCTCATGTTTGCCCGAGATATCATATTTGTGATTCTCATGGCTCTTTCCAGCACTTACATTCTCTCAGTTCTGCACAGACATGGAAAACGGATGCAAGGAAAAAGGCGTTCCAACAGAAGCCAGGAAATCACAGCAGAGAAGCAAGCAGCAAAAACTGTCGTGTTCTTTGTTACTTTCTATGTATTATTCTTTGGAGTAGAAAATACCGCATGGTTTATCCAGATTTCATTAAAAGAGGCTTATCCAGCACTAACTGATCTCCGCCATTTTATGTCCTTCTCTTTCCCATCTGCTTTTCCAGTTATTATGTTTGTACTGAATCCAAAAATTAGATACAAGCTCAAATGCAGCAGTATTAAAATGCATTCTGTGTCTGATTTGCACTCTAGGCACTACAGGAGCTAG

>*P. aethiopicus* *V1R166*

ATGATGGCAGTATATGACATCATCAAAGGTGCACTGTTTCTTGTGCTAGTAGTTATCGGACTCCCTGGGAACATAATGACAATGGCAGTATTTGTGGCTATACTAGTAAATGGCTCTAAACTCATTTTAACTGAAATAGTGATATCCAGTATTGCTGCAGTAAATTTTATCCTGATCATTACCCGAGGTCTTCCAGCAACCTTGCTTGTACTTTTTAATTTGAACAATTTGTATGGTGATACTGGCTGCAAATTTATAATTTATGGAGCAAGAATTTCAAGAAGTCTGGCAATTAATCTGACATGTGTTCTAGTCTGTATTCAGTGTATAACTCTTCTTCCTCCAACATCGAGTCTGTCATCGTTGAAACCAAGGTTGCCAAGATACATCCTAGTTGCATGCAGTTTAATTCTGGTATTAAATTTGTTAACAGAAGTAACCCCTGCTGTGTATACAGTATCAACATTAAATGCCACCACTCCACAGTATACATTTAATTTTGGGTATTGCATTGTCATTGCTCCTAATGCTGTTGTATTCTTTACTTTTGCTTTCACATATTTTATTCGTGATCTCTTATCAGTAATTATAATGACCCTTTCCAGTATGTACATTTTACAGATACTGTTTAACCACAGGAAACAAATATCCAACATAAAAAGTCAAGCACAAAGCCAGCAAACTGCTGTAGAGGTGAAAGCAGCAAAAGTTGTGGTCACTTTAGTAACTTTGTATATTCTTTTCATTGGATTTGAGAATGCTGTATTTCTGTACCAGATGACAGTCTCTCCGTATATACATCGTGTTATATCAGATGTTCGGCATTTTTTTTCTGTGTGCTATGCATCATTATTTCCATCTGTAATCATAGTGACCAATACAAGAATGCAACAACTTTTTAATTATTTGTATAAAAAAATGAACCTGCTAAATGAAGTATAG

>*P. aethiopicus V1R198*

ATGGATGGACGTGTTTGTTTATTCCCTTTGCTATTCTTACAGGAAGTACTCTATTTGACAGTAATGGAAGCATATGATATAATCAAGGGAATGGTATTCCTTGCATTAGTAGTAAGCGGCATCCCTGGCAACATAGTAGTCGCAATATCATTCATCTGTCTAACAGGAATAGGGTATAAACTGCTGCTAACCGATATTTTCATATGTAAAATTGCCATAGTAAATTTAGTACTGATTTTGACCAGGGGACTCCCAGTGACATTATTTGTTCTCTTCAGTTTAAAAAATCTCTACAGTGATGTCGAATGCAAAGTTATAATGTATTTAGCTCGTGTATCCCGTGGCATGGCTATCTGCTTGACTTGTATTCTTGTTTGTATTCAGTGCATTACTTTGAGTCCAACTACATCTAAACTGTTTGTCTTAAAACTCAGGTTGTCAAAGTATTCTTTAATTGCTTTTTATGTGATATTTGGAATAAATATGATAGCAGAAATTAGCCCACCAATGTACACTGTATCAAAGATAAATTCGACAAATCTGGAATATACTTTCCATTTTGGGTACTGTATAGTTCATTTTGCCGATTATACTCCATTTATGCTTACTGCAATTTCTTACATCGTTCGTGATTTTGTATTTTTAATTCTTATGGCAACTGCCAGTCTTTCTATTTTTCTGATTTTAATTAAGCACAAGAAACAAATTAAGGAGATAAGAAGTGATATGAAGAATCAATCCCAGTCAGCAGAAACAAAAGCAGCTAAAGTGGTGGTAACTTTGGCTTCATTATATGTCTTTTTCATCGGATTTGAGAATGCAATTTTTCTTTATCAGACTCTAGTCACAAAGAATCCAATTATTTCAGATGTCCGACATTTTTTTTCCGTTTGCTATTCCTCTATATTTCCTGCTGTTATAATTATAGCCAACAAACGAGTTCAAAATAGTATTAAATGCCGCTATGAATAA

>*P. aethiopicus* *V1R208*

ATGACTTCAGCCAACCTTGTTCAAGGCATAATGTGCCTTCTGATGACCGTCATGGGGATTTTTGGAAATATTCTAATTATGTGGTTGTTCTCCTACTTTGCAAACCTAAAATCAAAACTTACAACTATTGAAGTAATACTGTCAAACTTAGCAGGGGCTAACTTGGTGGTTGTGCTGACAGTAGGCATCCCGTATACTTTCATGGGTTTTGGGTTTCAAAATCTTTTTAACAATGCCGACTGTAAAAGCTTAAATTACTTATACAGAGTGTTAAGAAGCATGTCCATTAGCTTAACAAGTCTGCTTAGCTGTTTCCTGCTTGCAGCTATTAAGGCAACGAACTCTAAATGGGCTAATATAAAGTTTAAGTTACAAAAATACCTTCTACACATATTTGTTATTTTGTTGATAATTAGTATGGCCTTCAATATTGATGTTCCTCTTTTTGCATATGCTAAAACAAACTTAACAGGTGTAGATTTTACAGTAAACATGGGTTACTGTACTTTTCTGTACCCTGACAAATTTTCATTCCAAGCTGTATTTTACATCCTTTTCTTTGTAGACCTGCTGTTTGTGTTTCCTATGGTAGTGGCCAGTATACACATTTTAATAACTCTGTACAGGCACAGACAAAGAATAAAAAGTATTCGAAGTTCAGATCGAAACCAAGAGAACAGTGCAGAAGCCCAAGCAGCAAAAACTGTTATTACATTAGTAAGTCTTTATTTCTCCCTTTTTGGTATAGATAATGGATTCTGGATTTACCAGACAGTCAGTAAAGATGATCGCCAATACATGTCAGACATCAATCATTTCTTTACTGTTGGATACACTGCAGTATTTCCTATCATTATTTTTGTTTTAAATAAAAAAGCACAAAATAAGCTGAGATGCATTGACGTCTCCCAAGTTAGCTTGAGTCAAAATATTTCTGGTGCTGATTTTTCTATGCCCTGCTCACCAATAGTGTTCAGTCTTGCACAAGATTGTTCTCTACAGAACTTACCTATTCATTTATTTTTGCCCTGGAATAGTAAACTGGTGACTATTTATGTAACAAGAGCCACCATGCGAGTGGCAAATCTGTGGGACAGACTCAGGTGTGTGCAGCACTTAGGTGTATGA

>*P. aethiopicus V1R213*

ATGGATTTCTATGATTTCTTAGAGGGAATAGTGTGTTTCCTAATGACTTTGTTTGGAGTGGTGGGAAATATTATTACAATTTCATCATATGTTCAGATTGCACTTCAAGATGGAAGACTTACCACTGTTGAAGAATTACTGTCAATCCTAGCTGCAGCAAATATGGTTGCTTTGCTGTCACGTGGAATACCTGATTCTATGTTTGCATTAGGATGGGAAGGCACACTTACAGATATGCTATGCAAGGTTATTTCCTACATCCATATGTCATCCAGAGGACTAGTTATACGTTTAACATGCCTTGTTAGCTGTTTTCAGTACATAACATTAAGAACTACGTCCTCCAAATGGTCATTTTTAAAAACAAAACTGCAAGCATATATTTTGCCTGTCATTGTATGCCTTTGTCTAATAAGTTTGGCTTCTAGCCTAGATATACTGTTGTTTTCTGTTTCAGATTCTAATTTTACAAGCTTAGAAAATGCTTATCGTATAGGACACTGTGTCAATGCATTACCAAGTAAGTTAATATTTGATGCTGTTGGGTACTTTTTTTTCAGTAATGATCTTGTTGTTGTGATACTTATGATGTTATCCAGTGTACGAATCCTGCTGATGCTATACAGGCACAAGAAGAATGTCAGCAGTATCCGAAGCTCAGATCGGAACTTGAAAACTAGTGCTGAGAGCCAGGCTGCAAAGTCGGTTGTAACCGTAGTTATTCTGTATGTATGTTTTTTTGGAATTGGAACCATAATATGGTTTCATCAGACTATCACTAACTTAAGCATTGGTTTTGTTTCCTATGTACCAGATATACTATCTCTATGCTATGCT

>*P. aethiopicus* *V1R218*

ATGGAACGCTACAGCTTCACAAAAGGGGCCATATTTTGCATTATGGCATCGGTAGGACTTGCTGGAAACAGCATCATTTTGATTTCACTCTTGAGCAGTAAATACTATGAACACAAGTCTGCCCCAACAGAAATTATCCTAATATACACTGGCTTGGCAAACCTGTTTATGATCCTGTCACGAGGAGCTCCACATTCATTGTTTGTTTACGGAATTAGAAATCTCTTCAATGATGCTGGCTGCAAAGTTATCATCTATGTATCTAGGGTTTCTAGAGCCATGACAATATGTTTAACTTGTTTCTTAAGCTGTTTCCAGTGCAGTACCATTGCTTCATCCAGGTGGAAATGGGCTTACATAAAGGTCTACATGCAGACCTGTCTTGTACGTATTATGGTTGGTCTTCTGGTAATGAACATGGCGGCCTGCATTGCAGCACCGTATCTGTCAGTGCCTCTGAATAACAGTACTGAACATAAATACACATTTAATTTAGGATACTGTCTTGTTATTTTCCCCGATAATATTTCCTTTCAGGTGAACGGCTTTGCTCTGTTTGTAAGAGACTTGTTTTTTGTTGTTCTTATGAGTGTTTCAAGTGCATATATTTTATTGATTTTATATAAGCATAAGACAATGGTAAAACACATTAAAAACTCAGTTCAAAATCAACAGACTGCCGAGGGTCAGGCAGGTAAGACAGTAGTTACATTAGTATCACTTTATGTATTTTTCTTTGGGATAGACAATTCAATCTGGTTTTACCAGATAGCCTCTGGACTTGTACATCCGGTTGTCTCAGATGTCAGATTTTTTTTCTCTGTATGCTATTCATCTGCGTTTCCCATAGTAATTATTTCGCTGAACAAGAAAGTACGAAACAAGCTAAAGTGTACCAGGCAAGTACAACATGCATTGTTGCAG

>*P. aethiopicus V1R227*

ATGGACATCTATGACATCCTGAAGGCACTTATATTTTTAACTCTAGCCATTACTGGAATAACAGGAAATGGCTTTATTATACTAAGCTTCTGTGAAACTATCTGCCAGAAACAACATAAGAAACTGAAGACTACTGATATAATCATTGCGCAGCTAGCAACTTCAAATTTCATCATGGTACTTTCAAGAGGGGTTCCAGACTGTTTGTTTGCATTTGGCATCAAAAATCTCTTTAATGACACCGGCTGTAAATTTATTGCATTTATTTCCAGAGTAGCCAGATCCATGTCTATGTGTTTAACAAGCCTGCTTAGTTGTTTTCAGTTTGTCACTCTTGCCTCATCAAATGCTAAATGGGCATATATAAAAAACAGAATTCAAACATTTCTTTTAACTATAGTTATTTCCCTGTTGTTTGCAAATACACTGGCATATGTAACTGGAACTATTTTCTCTGTATCTGGAAGCAACTCCACTGATCTCAGATACTCATATAATTTTGGATACTGTCTTGTAACATTTCCTAGCAAAGAATCATTCCAAGCTAAAGGTTTTTTAACTTTTGCACAAGATTGTTTTGTTGTTGTTCTTATGGCTATGACTAGTGGTAGCATGCTATTCATTCTTTATTGGCATGGGAAACAGGTAAAAGGGATTAGAACTTCAGACCAGAGTCAAGAAGTAACAGCAGAGAACAGAGCAGCCAAAGCTGTTGCCATTCTTGTGAGTTTATACATCTTTTTCTTCGGGGTAGATAGCACGATTATGCTTTACCAAGTAACGTTTTTCCGAATTCATATCATTGTGTCAGATATTCGAACATTCTTCTCAGTATGTTATACTTCAGTCTTTCCATTCCTGATAATT

>*P. aethiopicus V1R257*

GGAAATCTGTTTATTAGTGCATCATTCTCTGAGATCAAATACCAAGAAAGTAAACTGACAACAGTTGAGACTATACTGTTAAACATTGCAATTTCAAATCTCATAATCTTATTTTCAAGAGGAGTTCCCGATTCTGTATTTGTATTTGGTTTACAAACAAAGTTCAGTGATTTCCTCTGCAAAGTTTTATGTTTTTCATTGATAACCTCCAGAGGGTTATCCTTAGCACTAACCTGCCTTCTGAGTTGTTTCCAATGTGTAACAATTGCAGTTTCACAACCTTCCATTATTAGTCTTAAACTGTATATTCAAAAACACATTTTGTCAGTAATCCTGTCTCTCTGTGTGCTTGCTATGGCATCCAGTACTGACTTATTAATATATGCAGTCTCAAGAACAAACCTTACCTCAGTGGCAAATGCATTTGATTTAGGATACTGTATTAATATGTTGCCCAGCAAGATTGTTTATGACCTCATAGGATTTGCGATTTTCAGTCGAGATCTTCTGTTTGTTATTGCCATGGCTCTAGCTAGTTTGAATATTCTGATATTGTTGTACAGGCACAGGCAAAATGTAAAAAGTATTCGAAGTTCAGAGAGACACAAAGGGCCCTCTGCTGAGGGCCAAGCAGCAAAAACAGTTGTAACTCTTGTAATTCTTTATATTTCGTTTTATGGAATTGACAATTCAGTTTGGCTGTATCAGATAGTGTCAAAGGAAAACATTAATGTTGTCTTTGATGTCCGCAAATTTGCTTCTGTGGGGTATGCTTCTTTCTTTCCCATTGTCATTGCAGCATTCAAC
