# Supplementary material for: Type 1 vomeronasal receptor expression in juvenile and adult lungfish olfactory organ
Source: Zoological Lett. 2023 Mar 10;9:6. doi: 10.1186/s40851-023-00202-z (PMC9999545; doi:10.1186/s40851-023-00202-z)
Supplement: Supplementary file 3 — Additional file 3: Supplementary Data S2. Nucleotide sequences of L. paradoxa V1Rs. [file 40851_2023_202_MOESM3_ESM.docx]

>*L.paradoxa* *V1R20* (*ancV1R*)

ATGGACCTGCCATACACAGTCCTTGAAGGGATCATCTTCATCATTTTGTCTGTGATTGGCTTGCTTGGCAATGGAATACTAATTCATGGCACACTAAAAAGCTTTGTGGGAAGAGTGCGAGCATCATGTGCAGTGCTGTTCTGTCTTGGTGTCATTCATATGTTCAGGATTTTGGTAGTGAACATACTGAGCATGATTTACAGTGTTGGAGGTGTAAAGATATTTGATGCTGCTGGGTGCAAAATTTTCAAGTTTACCTCTGCTGTCACAACTACTCTCTCCATCTGGTTTACCCTGTATGTGGCTGCATTTTATTACTTGAAACTCAGTCATGTGGTCCACCCTTTAAGTTTAACACCTACTGTTAGCTGGCGCAACAGGCATTTGCTGGGACTTTTTTCTCTTTGTGTAGCTGGACTTGCAGTCTATATTCCTATTTTAATTTACAGTGGAAAAGTCAGTGATTCAGACATGGCAAACACTACCAGTGCTCAGAAGCATGCCAGTTTGGTGTATGCTGCCTGCCAGATTAAGTATACCAGTCACAAGGTAGAACTTATCTATGGAACTGTGCTGTTGCTTTCCATTGATTTAGTGCCGCTGGCAGATTTTGTTATTATTAGCATACGAATAGCACTGCTGTTGTGGCAGCATCACAGAGCCAGCTATGGAGACATCTGGATTGATGCTGACAAGACAGAGACAGAAGTCTTCAGAGCAAGTAAACTTGCTGTGTTGTTAATGGGTCTTTCAGCTTGCCTCTGGGTGTCCCATTTTATTTTGTATCACTACCAATATGAACTGAGTTCCTGCTATTTTATTCCTGCTATACTTGCAGTGTTGTCGTCTGGCTATTCTTCATTCAGTCCTTATATACTCATTGTAATAAATTACAAAGTAAGGGAAAAGCTAAAAGACCTCTGCTGTCGGCATGCCATGAGGAATCCCCAAACGCAGAAAGTTACTGTTTCAGCTTCGCCATATTCAGAATGA

>*L.paradoxa* *V1R59*

ATGCAACTAAGCCTTCACACAGAAGGAGTGATATGCCTCCTTTTGACTTTGCTTGGAACTGTTGGCAACGTTGTTGTGATAGGGGCAGTTCTACCAATGAGCTGCCCCAAACTGAGACAAATGACTGTTGAAGTAATGTTGTCATGTTTGGCAGGGGCAAACCTGGTAGCTGTTTCAGTATCAGGAGTTCCTTTTTGTTTGTCAAGTTTAGGCTTTGAAAATCTTTTCAGCAACAAGTACTGTAAAGCTTGTACCTTTTTACACAGTGCATTTAGAGGTATGTCACTCAGTGTAACATGTTTACTTAGTTTGTTTCAGTTAGTAACTATTAAAGTAACCTCTGTAAAATGGACAAATATAAAACTATGCATGCAGAAATATATTCTACATATTCTTGTTTTACTCTTAGTAATAAATATATCTTTCTATGGAGTTTTATCAGTGTTTGCAGAGTCCAGATCAAACACTACTAAAACACTTGATAATCTTGAAGTAGGATACTGTACCTTTAAATATTCTAGCAAGCAATCCTCTGAAACTGTATTTGATATTCTTTTTGCACTGGATCTTACATATGTGATTCCTATGGCTTTGGCAAGTCTAAGCATTTTATGGATACTGCAAAAGCACAGACAGCAGGTTAAACATATTCGCACTTCAGCTAAAGCACAAGAAACTAATGCAGAGACTCAGGCTACGCGAACTGTTGTCACCTTAGTTACCATTTATGTTTCACTTTATGGAGTTGACAATTATTTCTGGGTCTCTCAGGCAAACACAGAAGATACTGGAGGTGATGTTCCATTCATGTTTAGTTTGTCCAACTTTCTTACTAACTCATACATGGCAGCCTTTCCTATTGTCACAGTTGCTCTAAACAAAAAAGTTAGAAATAAACTGAAATGCTGTCGTACATACAATTCAAATTAA

>*L.paradoxa* *V1R60*

ATGAAACCTTTTATTACAATGCAACTAAGCCTTCACACAGAAGGAGTGATATGCCTCCTTTTGACTTTGCTTGGAACTGTTGGCAACGTTGTTGTGATAGGGGCAGTTCTACCAATGAGCTGCCCCAAACTGAGACAAATGACTGTTGAAGTAATGTTGTCATGTTTGGCAGGGGCAAACCTGGTAGCTGTTTCAGTATCAGGAGTTCCTTTTTGTTTGTCAAGTTTAGGCTTTGAAAATCTTTTCAGCAACAAGTACTGTAAAGCTTGTACCTTTTTACACAGTGCATTTAGAGGTATGTCACTCAGTGTAACATGTTTACTTAGTTTGTTTCAGTTAGTAACTATTAAAGTAACCTCTGTAAAATGGACAAATATAAAACTATGCATGCAGAAATATATTCTACATATTCTTGTTTTACTCTTAGTAATAAATATATCTTTCTATGGAGTTTTATCAGTGTTTGCAGAGTCCAGATCAAACACTACTAAAACACTTGATAATCTTGAAGTAGGATACTGTACCTTTAAATATTCTAGCAAGCAATCCTCTGAAACTGTATTTGATATTCTTTTTGCACTGGATCTTACATATGTGATTCCTATGGCTTTGGCAAGTCTAAGCATTTTATGGATACTGCAAAAGCACAGACAGCAGGTTAAACATATTCGCACTTCAGCTAAAGCACAAGAAACTAATGCAGAGACTCAGGCTACGCGAACTGTTGTCACCTTAGTTACCATTTATGTTTCACTTTATGGAGTTGACAATTATTTCTGGGTCTCTCAGGCAAACACAGGAGATAAAGGAGACACAGGAGATACAGGAGGTGATGTTCCATTCATGTTTAGTTTGTCCAACTTTCTTACTAACTCATACATGGCAGCCTTTCCTATTGTCACAGTTGCTCTAAACAAAAAAGTTAGAAATAAACTGAAATGCTGTAGTACCCACAATGCAAATTAA

>*L.paradoxa* *V1R64*

ATGGAACTCCTCCGTTTTATCCGGGGAATTACAATTCTCCTATTAACAGTTACTGGAGCTGTGGGAAATCTCATCGTCGTATGGTCTTTCTTCCATATGTCCTATAAAAAATATAAACTCCTCCCTGTAGAAATAATCCTATGGAACTTGTCAACAGCAAACCTGATTGTGATTTTAATTGGAATCCCATATTCTCTTTATGATTTGGGGTGGAAAAATTTCTTCAGTGACAATGACTGTAAAATTTCACTCTTTGTATACAGGATTTCTAGAAACATGTCAATAAGCTTAACATGTCTTCTGAGTTGTTTACAAAATGCAACCATTAAAGCAACTATTAGCAAATGGGACAAGCTGAAACAGACTATACAGAAATACCTCCTTCATATATTTATATTGCTTTTGTTTATAAGTATGGCATTTAATTTGGATGTACCACTTTTTGCATTGTCAACGTCTAATGAGACCAACATTGATTATGTTGTACATTTAGGATATTGTACACTATGGTATCCTGATAAAACATCATTTGAAGCAGTATTTTATATTCTTTTTGCCCTTGACCTTGTATTTGTGATTCCTATGGCATTAGCAAGCCTGAATATTTTAGTGATTTTGTACCAACATAGAAAGCAAGTAAAAGGTATTAGGAGTTCAGACCAAAAGCTGGAGAACAGTGCAGAGAGTCAAGCAGCAAAGACAGTTGTTACATTAGTCATTCTCTATGTTACACTGTTTGGGATAGACAATACATTTTGGGTGTATGAAACAGTCAGCCATGAGGTTTATCCTTTTATGTTAGATATCACTCACATTCTTACAATATCATTCCTAGCATCATTTCCTGTTGTCATAATTATGCTAAAAAAAAATCCAAAACACAATTGTTTTGCTTCTTCAACTGAAGAGATGCACCAGATTCTGAGGAAAAAAAAAACGGTT

>*L.paradoxa* *V1R65*

ATGGAACTCAACTTTTTTATCCAGGGAATTATAATTCTCCTATTAACAATTACTGGAGCTGTGGGAAATCTCATCGTCGTATGGTCTTTCTTCTACATGTCCTATAAAAAATATAAACTCCTCCCTGTAGAAATAATCCTACAGAACCTGTCAACAGCAAACCTGATTGTGGTTTTAATGATTGGAATTCCATATTCTCTTTATGATTTGGGGTGGAAAAATTTCTTCAGTGACAATGACTGTAAAATTTCACTCTTTGTATACAGGATTTCTAGAAACATGTCAATAAGCTTAACATGTCTTCTGAGTTGTTTACAAAATGTAACTATTAGCAAATGGGACAAGCTGAAACAGACTATACAGAAATACCTCCTTCATATATTTATATTGCTTTTGTTTATAAGTATGGCATTTAATTTGGATGTACCACTTTTTGCATTGTCAACGTCTAATGAGACCAACATTGATTATGTTGTACATTTAGGATATTGTACACTATGGTATCCTGATAAAACATCATTTGAAGCAGTATTTTATATTCTTTTTGCCCTTGACCTTGTATTTGTGATTCCTATGGCATTAGCAAGCCTGAATATTTTAGTGATTTTGTACCAACATAGAAAGCAAGTAAAAGGTATTAGGAGTTCAGACCAAAAGCTGGAGAACAGTGCAGAGAGTCAAGCAGCAAAGACAGTTGTTACATTAGTCATTCTCTATGTTACACTGTTTGGGATAGACAATACATTTTGGGTGTATGAAACAGTCAGCCATGAGGTTTATCCTTTTATGTTAGATATCACTCACATTCTTACAATATCATTCCTAGCATCATTTCCTGTTGTCATAATTATGCTAAAAAAAAATCCAAAACACAATTGTTTTGCTTCTTCAACTGAAGAGATGCACCAGATTCTGAGGAAAAAAAAAACGGTT

>*L.paradoxa V1R70*

ATGATGAATACATATGAAACTGAAAAAGCATTGTCATTTCTTACCCTTCTAGTAATTGGAGTTCCAGGAAATGTGCTCATTCTTGTCACCTTTGCTCAGATTACTTACTTTGATCACCATCTTCTTCCAAGCGATGTTATATTAACAAATTTAGCTTTTGCAAATCTTCTCCATGTTGTTGCAAGAGGAGTGCCACAAACTCTGTTTTCCTTTGGCGTGAGAACTACGTTTAACACTCTTGGATGTCAACTGATAGTTTTTATCTTTAGAGTTACTAGAAGTCTCTCTATTTGTCTGACTTTCTTGCTGAGTGCTTTTCAGAGTGTCACAATTGCACCTGCTGATTCCAGACTGTCTTCACTTAAACAGAAGTTGGCAAAAAATAACTTGTATTTTATTATATTCTTTTGGATTTTAAGTGGAACTACAAATTTTGCATTCGTTTATAGCACATCACAAACAAATTTAACTGTGTACCGCTTTACTGTTAACTTGGATTATTGTTTTGTACAATTTCCTTCAAAGCAGTCATATGAAAGCAATGGCTTTATGTACCTAATCAGAGATCTTATTATTGTGATACTCATGGCACTGTGCAGTGTCTATATTCTCTCCGTTTTATACAGGCACAGCAAAACTATAAAAGGTATTCGAAGTTCAGATCGAACACAAAGTGTCTCAGCTGAAGCCAGGGCAGCAAAATCTGTTGTTAGTCTAGTTGTGTTATATGTTATTCTTTTTGGCATTGATAACATGATATGGGTTTACTCACTAATGGTGCCTCAGAATGCTGTCATAGCATCAGATATGCGACACTTCATTTCTTCTATGTATGCAGCAGTTTGTCCACTTGTCATCATATTTTTCAATAAGAAAGTTAGCAGTAAGCTCATGTGCAAAAAAAGTTGTAAGCATTCCAGCTTGCTATCTAGTGTTCTAACACCATTTGTTCTTCTTTAA

>*L.paradoxa* *V1R80*

ATGGATGTGAGAGTAATACTGAAGGCAGCTGCTTTTATTTTACTTCTCCTCATTGGGATACCAGCAAACCTCACCATCATGACATCATTCATCATCTCTGTAATTTCTGACAATAAACTCATGCCAACAGACTTCATTTTTACCAAACTGTCCTTTGTAAATGTTGTAGTTGTCCTTGTGAGAGGAATTCCACAGGCTCTCACAGCTCTAGGAATTCAAAAGATATTCAATGATATTGGGTGTAAATTTGTTATCTTTACCTACCGTGTTTGTCGAGCAATGTCTGTATGTATAACTTCTGTACTGAGCATCTATCAATGTATTGTCTTCTTACCTCCTTCATCAAAATATGCAACACTGAAACAAAAGTTTTCTCAGAATATTTTTATTATCTTCATTTTTCTATGGTGTATCAACTGTATAATTTACATACCTGCTGGTTTTATGTATTCACAGTCAGAAGTAAATTCAAGTATTCCCAAATATGCTCTCAACTTAGAGTTCTGTTTTGTTTTATTCCCTCATGAAGTATCATATAGACTTAATGGAGCAGTTTATACCTTCCGAGATTTTCTATTTGTTGGACTTATGACACTTGCCAGCAGCTACATTGTTGCAATTCTATACAAACATAATAAAAAACTCCAACGTATAAGAAGTCCTGACCAAAAACAAGGAAGTGCCATAGAAGTAAGAGCAGCTAAGTCTGTAGTTATGTTGGTTACACTGTACCTTGTTTTATTTGGAGTTGATAATGCCACCTGGATGTATACTGTCATGGCTGCTAATGTGGAACCTGCAGTTTCTGATGCTCGTACCTTCTTTGCCTCATTGTATACTGCAGTTAGTCCTGTGATAATAATTGGAACAAATAAAAAAATTCAACAAAAACTGAAATGTACTTCTTCAGGCAGTGAAAATAAAGTAAAGAAGACTATTGTGACACATAATGATATCACAAAAAATACTGCATCTTAA

>*L.paradoxa* *V1R89*

ATGTGTAAAGGAATATTGTTCTCACTGATGACATCATTTGGAATTATTGGAAATATGATTACTATATCCTTATTTTTGCACATTGTTTACTTTAAAGGAAAACTGTTAACTGTTGAGATAATATTATCATTGCTGTCCGGAGTAAACCTAATCATGTTATTATCTAGAGGCATTCCAGATCCACTATTTGTATTTGGAGTAAGAAATGTTTTCAATAATATTGCTTGCAAAATAGTGACATATATACATATATGGTTTAGAGGACTAGCTGTAAGCTTAACCTGTCTACTAAGTTGTTTTCAGTGTGTAACAATTAGTGTTAATTATAATAATCTGGCAAATTTAAAGAGCAATTTACAAAATAAATTGTTATTTGTCGTTATTTTTCTCTGCCTAATAAGCATGGCTTCCAGTATTGACATAATATTATATTCAGTTTCAGGTGCTAACTTTACAAGCTTAAAAAATACAATTCATAATGGCTATTGCCTCAACATTTTACCCAGCAAACTTGTTTTTGATACCATAGGTTACATGATATTCACACGTGACCTCATATTTGTACTTCTGATGTTCCTCTCGAGCTGTAATATCTTATTAATTTTGTATAGACATAAAAAAAAGATAAGTAGTATACGAAGCTCAGATCATAATTCAAAATGCAATGCAGAAAGCCAAGCAGCCAAAACAATTGTAATTGTGGTTATCATGTATGTCTGCCTGTTTGGAATTGGCAGTACAATCTGGTTTTACCAGTCTGTGTCAGAAACAAAAAGTGATATTCTTTCTAATGTACCTGATTTTCTTTCTATGTGTTATGCATCACTTTTTCCTGCTGTTATAATTATGTTCCATAAAAAGACTCAAAATGTGCTGAAACACTGTTTAAATAATGAAGATTTTCAAAAGTAA

>*L.paradoxa* *V1R92*

ATGGAACTAAATCGCATCATAAGAGGAATCCTTTTCATTATAATAGCACTTACTGGAATCCCTGCTAATATTGCAGTTGTGGGATCAGTGTCCTGGACAGCATACCATTCTTTTAAATTACTGCCATCTGAAGTTGTAATCTGTAACTTAGCTTTAGCAAACACAGTCTTAGGATTCACCAGGGGACTCCCAGCAGCCCTTTTTATGCTCTTTGAGATGACAGTAAATTCGAATATTGGCTGCATTGTTATAGTTTACTTAGCCAGAATATCCAGAGGACTGTCAATCTGTTTCACATGTGTGCTTAGCTGTGTCCAGTACATAACTATTATTCCAGCTACATCCAAGTGGGGTTATTTTAAATGCAAAGTGACAAGTCATATTTTACTTCTGGCATTTTTGCTTTGGCCATTGTACATTATTATGGAAAGTTCTGCTATTTTTGTGGCACGTGCAGTAAAGAACTCCACATACTCAGAGTTTACATTTAATTTTGGCTACTGTCTGGCTGTATTTCCCACTGAACTCACATATCAGTTAAATGGATTTGGTCTTTTTGGCCGTGACCTCATAGTAGTAGTTGTGATGACAGTTGTTAGTGTATACATACTGGTAATTTTGTTTCAACACAGAAAGCAGGTAAATAACGTAAGAAGCAACAAAGCAATGAATCAAGCTGCAGAACTTCGTGCAACTAAAACTGTAGTGTCCTTAGTAATAATGTACATTTTCTTTTTTGGGGTTGAAAACAGCATCTGGTTTTACCAGATCATTGGTTCAAAAGTTGTCCATCCTGTGGTTTCAGATGTTCGTCATTTCTTTTCTGTGTGTTACACAGTCTTTTTTCCCACTGTAATTATTGCATCAAATAAAAAAATACAACTTGTCCTAAAATGTTTTTATTGCCAGCCAAAGCCAGTTACAGAATTTACCTGCAGTACATAA

>*L.paradoxa* *V1R93*

ATGGAACTAAATCGCATCATAAGAGGAATCCTTTTCATTATAATAGCACTTACTGGAATCCCTGCTAATATTGCAGTTGTGGGATCAGTGTCCTGGACAGCATACCATTCTTTTAAATTACTGCCATCTGAAGTTGTAATCTGTAACTTAGCTTTAGCAAACACAGTCTTAGGATTCACCAGGGGACTCCCAGCAGCCCTTTTTATGCTCTTTGAGATGACAGTAAATTCGAATATTGGCTGCATTGTTATAGTTTACTTAGCCAGAATATCCAGAGGACTGTCAATCTGTTTCACATGTGTGCTTAGCTGTGTCCAGTACATAACTATTATTCCAGCTACATCCAAGTGGGGTTATTTTAAATGCAAAGTGACAAGTCATATTTTACTTCTGGCATTTTTGCTTTGGCCATTGTACATTATTATGGAAAGTTCTGCTATTTTTGTGGCACGTGCAGTAAAGAACTCCACATACTCAGAGTTTACATTTAATTTTGGCTACTGTCTGGCTGTATTTCCCACTGAACTCACATATCAGTTAAATGGATTTGGTCTTTTTGGCCGTGACCTCATAGTAGTAGTTGTGATGACAGTTGTTAGTGTATACATACTGGTAATTTTGTTTCAACACAGAAAGCAGGTAAATAACGTAAGAAGCAACAAAGCAATGAATCAAGCTGCAGAACTTCGTGCAACTAAAACTGTAGTGTCCTTAGTAATAATGTACATTTTCTTTTTTGGGGTTGAAAACAGCATCTGGTTTTACCAGATCATTGGTTCAAAAGTTGTCCATCCTGTGGTTTCAGATGTTCGTCATTTCTTTTCTGTGTGTTACACAGTCTTTTTTCCCACTGTAATTATTGCATCAAATAAAAAAATACAACTTGTCCTAAAATGTTTTTATTGCCAGCCAAAGCCAGTTACAGAATTTACCTGCAGTACATAA

>*L.paradoxa* *V1R99*

ATGAATAATGCAGATATTAGGGCAGGCCTCTCGTGGCAAACTCAACAGTGGAGACTGGTAATTGTGTGTATCAGGTCTCTCTGGGGACTGAAGAGAACCAATGTCATACTTGTCAGACCATTATCAAGTTCTGTTTATAACGTGACATATCTGACAGGCCTAAGAGCAGACCAGTTTGCGTCAACACTTGCCCATCCATCAAGAATGAGTCTGTACGAGCACATAAAAGGAGCTGCACTATTTGTAATGACTTTGTTTGGATCCTTTGGCAACATAGCTACAATTTTGTCATTTATGCAGATTGCATACCAGGAAAGAAAGATTCTACCTGTTGAAATAATTCTGTCAAGCCTCTCAGGAGTAAATCTTTTGATATTACTTTCACGAGGAATACCTTATCCTCTGTTCATATTTGGTGTGATTGTATCTTTTAATGACCCAGCCTGCAAAGCTGTTTCTTACATACACATATGGTTTCGGTCACTTGGTGTCAATTTAACTTGTCTTCTAAGTTGCTTTCAGTGTGTGACTATTACCTCTAGTACTGCAAAATGGGTCACACTAAAAGGAATTTTACAGAAACATCTTTTAACTCTTATTAGCTTTCTCTGCTTAGTTAGTATGGCATCAAGTGTGGACATCATTTTGTTTGGATCCTCTGGCACTAATGTTACAGGATTGCAGAATACAATTTTTAATGGATACTGCCTCAACACTTTGCCTAGTAAGATTGTATTTGACACAATTAATTATCTTATTTTTGCTCGTGATGTTGCATTTTTGCTTCTTATGACTTTGTCTAGCTGTGTTATTTTAATAATCTTGTATAAACATCAGAAGAAAGTAAGTGGCATAAGAAGTTCAGAGAAAAACTTAAAAACTACTGCAGAAGGGCAGGCAACTAAGACTGTTGCCATTATAGTCGTCATGTATGTGTTCTTCTTTGGTATGGGCACCACAATTTGGTTCTATGAAGCAGTAGCTGATACTAAGGTACATTTCCTTGCAGTGATACCTGATTTTCTTTCTGTATGCTATTCTTCCTTCTTTCCTATGGTTATTATTATGTTTAATAAAAGAATTCAAAATGTACTTAAGGACTTCTTCAGTAAGAGTGAAAATAAAGTTGATGAATTATCCACATGCAGACACATAATGTTAAAATCATATATTACCAGTTCTGCAATATTTTACAAACTAAATTATTTCCAAAGTTTTCAGAATTATTTAACAAATGATATACAGAACATGTTCGCTGGGTGTCAGCTGATTAAATTCTCAGCTTTTTTTGTCAAGGCAGTCTTTACCTGGGACAGTGGTATTTAG

>*L.paradoxa* *V1R103*

ATGGAAGTATATGCCATCATTAAAGGACTTCTGTTTTTGCTGATAGCAGTTGTTGGAATTCCTGGAAACTTGGCCATCACTGGATCCTTTTCTTGGATCGCTTACTCTGTCATTAAGCTACTGCCGGTTGAACTAATTATATGGAATTTATCACTTGCAAATGCAATACTAGTTTTTACCAGGGGTCTTCCTGCAGCACTTTTTATGCTATTTGGAAGAAAAGTGAATAGCGATGCAGTCTGCAAAATTATAATTTATCTATCCAGGATATCAAGAGGCATGGCATTTTGTTTAACTTGTGTACTCAGCTGTGTTCAGTGTGTAACTCTAATTCCACCATCTTCAAAATGGGCTCATATAAAACAAAATGTGCCAAAGTATACTTTTCCTGTCACTTTTTGTCTATGGTTGCTGTATGTGATAATGGAAATACACGGACTTAGTTTTTCAAGTTCTGAGACAAATTCCACAAATCCAGAATTGTTATTTAATTTGGGATATTGTGTTGTCATTGTTCCAAGTATCATTGCATTTTATGTTTATGGCTTTCTCTTTTTCAGCCGAGATCTCATGGTGGTAGGTCTTATGACTATAGCTAGTGCCTACATTTTACTGGTTTTGTTTCGACATAGGAAACATGTGACTAACATGAGGAACCCTAACCACAACAGTGATGTGGAGATCAGAGCAGCTAAAACTGTTATATCCTTAGTCACTATGTATGTCTTCTTCTTTGGGGTCGAGAACACTATATGGCTTTATCAAATAGCAGTTTCAAAAATTATTCATCCTGTAGTTTCTGATGTTCGGCATTTCTTTTCTATTTGCTACACCTTTTTTTTCCCCATCTTAATAATTGTGTCAAATAAAAAAATTCTAAATGTTTTTAAATGTTTATGGAAACCATCTACTCATAATGACACTGTTTCTTCTCTTTCCTGA

>*L.paradoxa V1R104*

ATGGAAGTATATGCCATCATTAAAGGACTTCTGTTTTTGCTGATAGCAGTTGTTGGAATTCCTGGAAACTTGGCCATCACTGGATCCTTTTCTTGGATCGCTTACTCTGTCATTAAGCTACTGCCGGTTGAACTAATTATATGGAATTTATCACTTGCAAATGCAGTATTAGTTTTTACCAGGGGTCTTCCTGCAGCACTTTTTATGCTATTTGGAAGAAAAGTGAATAGCGATGCAGTCTGCAAAATTATAATTTATCTATCCAGGATATCAAGAGGCATGGCATTTTGTTTAACTTGTGTACTCAGCTGTGTTCAGTGTGTAACTCTAATTCCACCATCTTCAAAATGGGCTCATATAAAACAAAATGTGCCAAAGTATACTTTTCCTGTCACTTTTTGTCTATGGTTGCTGTATGTGATAATGGAAATACACGGACTTAGTTTTTCAAGTTCTGAGACAAATTCCACAAATCCAGAATTGTTATTTAATTTGGGATATTGTGTTGTCATTGTTCCAAGTATCATTGCATTTTATGTTTATGGCTTTCTCTTTTTCAGCCGAGATCTCATGGTGGTAGGTCTTATGACTATAGCTAGTGCCTACATTTTACTGGTTTTGTTTCGACATAGGAAACATGTGACTAACATGAGGAACCCTAACCACAACAGTGATGTGGAGATCAGAGCAGCTAAAACTGTTATATCCTTAGTCACTATGTATGTCTTCTTCTTTGGGGTCGAGAACACTATATGGCTTTATCAAATAGCAGTTTCAAAAATTATTCATCCTGTAGTTTCTGATGTTCGGCATTTCTTTTCTATTTGCTACACCTTTTTTTTCCCCATCTTAATAATTGTGTCAAATAAAAAAATTCTAAATGTTTTTAAATGTTTATGGAAACCATCTACTCATAATGACACTGTTTCTTCTCTTTCCTGA

>*L.paradoxa* *V1R120*

ATGACTGTTGACAGCATCACATCCCACCTTGCTTTTGTTAACATGATAGTTCTGCTAACAAGAGGAGTCCCACAGACAATGACTGCTTTTGGATTACAGAATATTTTAAATCACAGTGGCTGTGTCTTTGTTATTTTTGTTTATTCAACAGTAAGAGCTCTTTCAGTGTGTATCACTTGCCTTTTAAGCATATTCCAGGCTGTTACTATTGCTCCATCAACCTGCAGTTGTATCTGCCTGAAAATGAAAATCCCACAATACCTTATGCCATGTTCATTCATACTATGGTTAGCCAACATGGCTATTTGTGGTGGTCTGCTGGTTTATACAACTGTCCCTCAAAATGGAACAATTCCAAAATATACAATCAGTACAGGGTACTGTTATGTAAAATTTCCTGGTGAAGTTGTATATAATGTTTATGGAGCTGTATACACAGGGCGTGATGCTGTAATTGTATGTGTGATGGTAATATCAAGTGGTTATATTTTATTGACACTGTATAGACACAACAAACAAGTTAAGAGTATAAGAAGCTCAAATTCAGGTCCAAAAGCTACAGCAGAGGTAAAAGCTGCTGAAATTGTCATATGCTTGGTAATACTTTATGTCATGTTTTATGGAATTGATAACATCATTTGGATATACATGATAATTAAAACATCTGCTTCTCCTCAAGTTGTAGATCTGAGAGTGTTCTTCTCTTCTTGCTATGCATCTCTAAGTCCTTTTCTAATTATTAGTTTTAATAAGAAGATTCAACAGAATATTTGCATTAAAACTAGAATATAA

>*L.paradoxa* *V1R121*

ATGGCACCAGTGAATCTCATCATATCACACCTTGCTTTTGTTAACATGATAGTTCTGCTAACAAGAGGAGTCCCACAGACAATGACTGCTTTTGGATTACAGAATATTTTAAATCACAGTGGCTGTGTCTTTGTTATTTTTGTTTATTCAACAGTAAGAGCTCTTTCAGTGTGTATCACTTGCCTTTTAAGCATATTCCAGGCTGTTACTATTGCTCCATCAACCTGCAGTTGTATCTGCCTGAAAATGAAAATCCCACAATACCTTATGCCATGTTCATTCATACTATGGTTAGCCAACATGGCTATTTGTGGTGGTCTGCTGGTTTATACAACTGTCCCTCAAAATGGAACAATTCCAAAATATACAATCAGTACAGGGTACTGTTATGTAAAATTTCCTGGTGAAGTTGTATATAATGTTTATGGAGCTGTATACACAGGGCGTGATGCTGTAATTGTATGTGTGATGGTAATATCAAGTGGTTATATTTTATTGACACTGTATAGACACAACAAACAAGTTAAGAGTATAAGAAGCTCAAATTCAGGTCCAAAAGCTACAGCAGAGGTAAAAGCTGCTGAAATTGTCATATGCTTGGTAATACTTTATGTCATGTTTTATGGAATTGATAACATCATTTGGATATACATGATAATTAAAACATCTGCTTCTCCTCAAGTTGTAGATCTGAGAGTGTTCTTCTCTTCTTGCTATGCATCTCTAAGTCCTTTTCTAATTATTAGTTTTAATAAGAAGATTCAACAGAATATTTGCATTAAAACTAGAATATAA

>*L.paradoxa* *V1R127*

ATGCAACTAAACATTAACACAGAAAGCATTGTGTGCCTTCTTTTGACCTTATCTGGAGTGATTGGTAATGCAATTGTTTTAGGAGCCATTCTGCCCATGGTCTTCTCCAAAATGAAACAAATGACAGCGGAAATTATCCTGTCAAGTTTGGCAGGGGCAAACCTGATTGTTATTTCAGTATCAGGAGTTCCTTATTGTTTGTCAAGTTTGGGGTTTGGAAACCTCTTCTCTACCAGGTACTGCATAGCTGTTAGTTACTTGTTTTATATGTTTAGGAGTATGTCAGTCAGCTTAACATGTTTACTAACACTCTTCCAGTTGGTAACAATTAAGACAACATCTATAAAATGGGGAAAATTAAAACTTGTTATACAAAATTATCTTCCACAAATAGTTGTTTCACTGTTATTGGTAAACATGTGTTTTTATGTGTCATTACCAGTTTATGCAGAGTCCAGAACCAACACTACTGAAATGCATGTCAGTATCAAGGCAGGATACTGCATTTTCAAGTACCCTAATAAGCAGACATTTGAAATGATATTTTATATTCTTTTTATATTTGATCTTTTATTTGTAATTCCTATGACTGTGGCAAGTATAAGTATTCTACTAATACTGCAAAGGCACAGACAGCACATTAAAGGTATTCGAAGTTCAAGTCGAGCACAAGAAACCAGTGCAGAAAGTCAAGCTACAAGAATGGTTCTCACTTTGGTTACTGTTTATGTTACACTTTATGGAGTTGATGATTATTACTGGATCACTCAGACAAACGCAACAGGTGAGACCCCATTCATGCTTGATATGTCGAACTTTCTTGCCATTGCCTATGTGGCAGCTTTTCCTATTGTCACAGTTGCTCTAAACAGAAAAATTAGAAATAAACTCCAA

>*L.paradoxa* *V1R130*

ATGGATACTTATCGTATTTGTAAGGGAACACTGTTTCTCTTGCTTGCAGTTATAGGAATTCCTGCCAACATTGCCATCTTAGCATCATATGGAAGAATAGTCTGGTTCAAGAACAAGCTCTTTCCTGCTGAAAAGATTCTTTCTGCTCTAGCATTTGTAGATATAGTTATGATTCTGACACGTGGATTACCTTATACTTTATTTTCATTTGGAGCTGAGGATCTCTTCAATAAAGTAGGATGTCAAAGTATTCTGTATATATCCCGTGTTTCCAGGGCTATGGCAATTGGTCTAACCTGTCTTCTCAGCTGCTTTCAGTTGATCACAGTTTCCCCAGCAGCTTCAAGACTGGCGCAGATGAAAAGCAGTGCAACAAAGTATCTGGTAGTGATCATATTTTCCATCTTGCTTTTGAATCTGATCCTGTGTTACTGTTCTGTCACTTATGCAATCCCAAAGACAAACACAACAAACATGGAATTTGCCTTCAATCTTGGGTATTGTATCCTCAAATTTCCTAGTGAGGTTGCATATCATGGATTTGGCTTTATTCTTTTAGTACGAGACCTTTTCTTTGTAATACTTATGGGGCTTGCCAGCGCAGCAATCCTTTTTGTGTTATTCCAGCACAGAAAACATGTGAAGCAAATAAGGAATACTCGCTTTACCTCTGAATGTGACAGTGAATGGAAAGCAGCCAAAGCAGTTGTTGGTTTAGCTTCCATGTACATCTTTTTCTTTGGTGTAGAGAACACAATCTTTCTTGTCACCATGACTGGTAATAAGATCAATTCTGTTGTGTCAGATGTTCGCATTTTTTTCTCTATTTGTTACGCATCCGTTTTTCCTGTTGTTGTAATTGCAACTAATTCCAAGGTCAGAAAGCAATTAAACTGTGAAATTCAGGAGAATGAAACAGAAACATGCAGCACTTCCCACACAATATGA

>*L.paradoxa* *V1R139*

ATGGAAGCATATGATGTAATCAAGGGAACAGTATTTCTTGCATTAGTAGTCAGTGGAATTCCTGGCAACTTAGCAGTAATAATATCATTTGTCTGTTTAACAGTAATGGGGTTTAAACTGCTGCTAACAGAAATGTTCATATGTAAAATCGCCATAGTAAATTTCATACTGATTCTGACCAGAGGACTTCCAGTATCTTTATTTGTTCTCTTCACTTTAAAAAATGTCTACAATGACGAAGGATGCAAAGTTATAATGTATTTTGCTCGTGTATCACGCGGCATGGCTATTTGTTTGACTTGTATTCTTGTCTGTGTTCAGTGCATTACTCTGGCACCAACAACATCTAGACTGTTTGTCTTAAAACTCAAGTTGTCAAGGTATTCCTTAATTGCTTTCCATGTAATATTAGGAGTAAATATGATAGCAGAGATTAGCCCACCAATGTACACTGTATCAAAAATAAATTCCACAAATCTGGCATATACTTTTCATTTTGGGTACTGTATCGTTCATTTCCCTGATTATACTCCATTTATGCTTACTGCAATTTCTTATATTGTTCGTGATTTTGTATTTGTAATGCTGATGACAGCTGCAAGTTTTTCAATTGTCCGTATTTTGATTAGGCACAGGAAACAAGCTAAGGACTTAAGAAATGATATGAAGGATCGCACCGAGTCAGCAGAAACAAAAGCAGCTAAAGTGGTTGTAACTTTAGCTTCATTATACATCTTTTTCATTGGATTTGAGAATGCAATTTTTCTTTATCAGACTCTTGTCACAAAGAATCCACTTATTTCAGATGTCCGACACTTCTTTTCTGTTTGCTATTCTTCTGTGTTTCCTGCTGTTATAATTATGGCCAACAAAAGGGTTAAAAATTGTATTAAA

>*L.paradoxa* *V1R142*

GTTGAAATAATAATTCTTAACCTTGCTTGCTCAAACTTACTTATTTTACTTTCAAGAGGCATTCCTGATGTTTTTTTTGTCTTTGGTTTAAAGAATATATTCAGTGATAATTTGTGCAAGGCCATTTCTTTTGTACACATTAGCTTTAGAGCACATGCATTGTGCCTAACTTGTTTTCTCAGTACTTTTCAGTGTGTGAGTGTCAGTGCAACAAACTTTAAATGGCTTCAGTTAAAATTAAAAATGCAAAAGTATGCTTTAACTCTAATTGCTTTATTCTGCATTGCAAGCATGGTATCCAGTATAGATCTATTATGTTTTTCTGTCGCAAGGAAAAATGTTAATAATACAGAAACCTCAATCTCCTTAGGATACTGTATTAATATTTTGCCATCCACACTCTTATTAGATCTAATAGGGTATTTAATATTTGCTCGAGATCTTTCATTTGTGATCATCATGTGCTGTTTCAGTTTTTATCTTTTGTTAATCCTTTACAAACACAAACAGAAAGTTAAAGCTATTCGAAGCTCTGACCGAAGTCTGGAAACTACTGCAGAAAATCAAGCAGCAAAAACTGTTGTTACTTTAGTTATCCTGTATGCTTCATTTTTTGGAATAGGCACTACAATATGGTTTTACCAAGTTGTGTCTGAAACAAGTTTTAATGCAGCATCTATCATTCGAAATTTTTTCTCGATGTGCTATGCTGCATTATTCCCAATAGTTATTATAGCATTTAATAGAAAAATTCAGCAGAAACTGAAACAATGA

>*L.paradoxa* *V1R172*

ATGAAAGTAGCATGTGCAAATCTGATAATGGTTCTAACACGAGGATTACCTCAGTCTATATTTGTATTTGGATTAAGAAATCTGTTTAATACAACTGGCTGTAAACTAATTACCTTTTTATTCAGACTATCTAGAGCCATGTCCATCTGCTTAACATGCCTTATGAGCTGCTTCCAGTATGCCACGCTGGCCTCTACAAGTCCAAAATGGCTCTATTTAAGAAACAAAATGCAGACGTTCCTTTTTCCAATCACATTTTTTTTTATATTACTAAATACTTCAGTGTATATCACTGGAGCAGTTTTTTCAGAGTCTACTAAAAATACTTCTGAAATAAAACATTTTTACAATCTGGGCTACTGCCTAGTAATCTTTCCCAATCAAATGTCATTTCAAGCAAACGGTTTTACAATTTTTGCTAGAGACCTTATATTTGTCATCGTTATGGTTCTAGCTAGTGCAAACATTTTACTACTTTTATATTTGCATGGGAAACAGGCTACACAGATACGAAGTTCAAAACAAGATCAGGAAGCCACTGTGGAAAGAAAAGCTTCAAAGACTGTTGTCACTTTAGTTAGCTTATATGCATTATTCTTTGGAATCGATACCACCATCTGGCTTCATCAAGCATCTGTCTCCAACGAAGTGCAAATTGTGATATCAGACATCAGGAATTTTTTTTCCACGTGCTATGCTTCAGTCTTTCCCATTGTTATCATCAGTGTCAACAAAAATATTAGAACTAAAGTAAAATATTCTATAGGTAGACAACAAGCAGAATTAAAAGCTATATCCCTTATGCACAATATATAA
